# The effects of glucagon-like peptide-1 receptor agonists (GLP1-RAs) on alcohol-related outcomes: a systematic review and meta-analysis

**DOI:** 10.1186/s13722-025-00637-z

**Published:** 2025-12-05

**Authors:** Binayak Sinha, Samit Ghosal

**Affiliations:** 1 Department of Endocrinology, Manipal Hospital, Kolkata, India; 2 Department of Internal Medicine, Nightingale Hospital, Kolkata, India

**Keywords:** Glucagon-like peptide-1 receptor agonists, Alcohol use disorder, Alcohol consumption, Alcohol-related events, Semaglutide, Tirzepatide, Meta-analysis, Systematic review

## Abstract

**Objective:**

To evaluate the effects of glucagon-like peptide-1 receptor agonists (GLP-1RAs) on alcohol-related outcomes in adults with or without alcohol use disorder (AUD).

**Methods:**

A systematic review and meta-analysis following PRISMA guidelines searched PubMed, Embase, and Cochrane Library up to May 3, 2025. Eligible studies included randomized controlled trials (RCTs) and observational studies assessing GLP-1RAs (e.g., Semaglutide, Liraglutide, Exenatide, and Dulaglutide) versus placebo, no treatment, or other interventions in adults. Outcomes were alcohol consumption (defined as total intake or drinks per drinking day, measured as standardised mean difference [SMD]), alcohol craving (SMD), and alcohol-related events (hazard ratio [HR]). Random-effects models with Restricted Maximum Likelihood estimation and Hartung-Knapp adjustment were used. Separate AUD and SUD meta-analyses addressed outcome heterogeneity, with intoxication reported narratively.

**Results:**

Three RCTs (*N* = 430) and six observational studies (*N* = 2,740,207) were included. RCTs showed non-significant reductions in alcohol consumption (SMD: -0.24, 95% CI: -0.70, 0.23), drinks per drinking day (SMD: -0.23, 95% CI: -0.64, 0.19), and craving (SMD: -0.14, 95% CI: -2.84, 2.55), with Semaglutide showing greater craving reduction (*p* = 0.024). Observational studies showed reduced alcohol-related events (HR: 0.64, 95% CI: 0.59–0.69, *p* < 0.001), with separate analyses confirming effects for AUD (HR: 0.66, 95% CI: 0.63–0.70) and SUD (HR: 0.66, 95% CI: 0.18–2.48), and intoxication (HR: 0.50). Semaglutide and GIP/GLP-1RAs had more potent effects (*p* < 0.001).

**Conclusion:**

Observational studies suggest a decrease in alcohol-related events, but RCTs have effects on alcohol consumption and craving that remain non-significant. Larger RCTs are needed.

**PROSPERO ID:**

CRD420251045294.

**Supplementary Information:**

The online version contains supplementary material available at 10.1186/s13722-025-00637-z.

## Introduction

Alcohol use disorder (AUD) represents a significant global health burden, affecting approximately 5.1% of the adult population worldwide and contributing to 3 million deaths annually [1]. In the United States, AUD prevalence is estimated at 28.1 million adults aged 18 and older (10.9% of this age group), with substantial morbidity linked to excessive alcohol consumption, including liver disease, cardiovascular complications, and psychiatric comorbidities [2]. Current pharmacological treatments for AUD, such as naltrexone and acamprosate, have shown modest efficacy, with response rates—defined as the proportion of patients achieving abstinence or a significant reduction in heavy drinking—often below 50%, highlighting the need for novel therapeutic options [3].

Glucagon-like peptide-1 receptor agonists (GLP-1 RAs), such as semaglutide, liraglutide, and dulaglutide, as well as dual agonists like tirzepatide, are primarily used for managing type 2 diabetes mellitus (T2DM) and obesity due to their effects on glucose homeostasis and appetite regulation [4]. Recent preclinical studies have suggested that GLP1-RAs may modulate reward pathways, reducing alcohol-seeking behaviour in animal models by acting on the mesolimbic dopamine system [5]. Emerging clinical evidence supports these findings, with observational studies indicating reduced alcohol-related events in patients treated with GLP1-RAs for T2DM or obesity [6]. Additionally, randomized controlled trials (RCTs) have explored GLP1-RAs’ effects on alcohol-related outcomes in AUD populations, showing potential reductions in alcohol intake and craving [7]. However, the evidence remains heterogeneous, with varying study designs, populations, and outcomes, necessitating a comprehensive synthesis to evaluate the efficacy and safety of GLP1-RAs in this context. To address this knowledge gap, we conducted a systematic review and meta-analysis using the PICO framework: in adults with or without diagnosed AUD (**P**opulation), does treatment with GLP1-RAs (**I**ntervention) compared to placebo, no treatment, or other pharmacological interventions (**C**omparison) affect alcohol consumption, AUD incidence/recurrence, and alcohol craving (**O**utcomes)? This review aims to provide evidence to guide clinical practice and inform future research on GLP1-RAs as a potential treatment for AUD.

## Methods

This systematic review and meta-analysis were conducted in accordance with the Preferred Reporting Items for Systematic Reviews and Meta-Analyses (PRISMA) guidelines registered with PROSPERO (ID: CRD420251045294) [8, 9], and a completed PRISMA checklist is provided as a “related file”. The protocol was developed to evaluate the effects of glucagon-like peptide-1 receptor agonists (GLP1-RAs) on alcohol-related outcomes in adults.

### Eligibility criteria

**Fig. 1:**
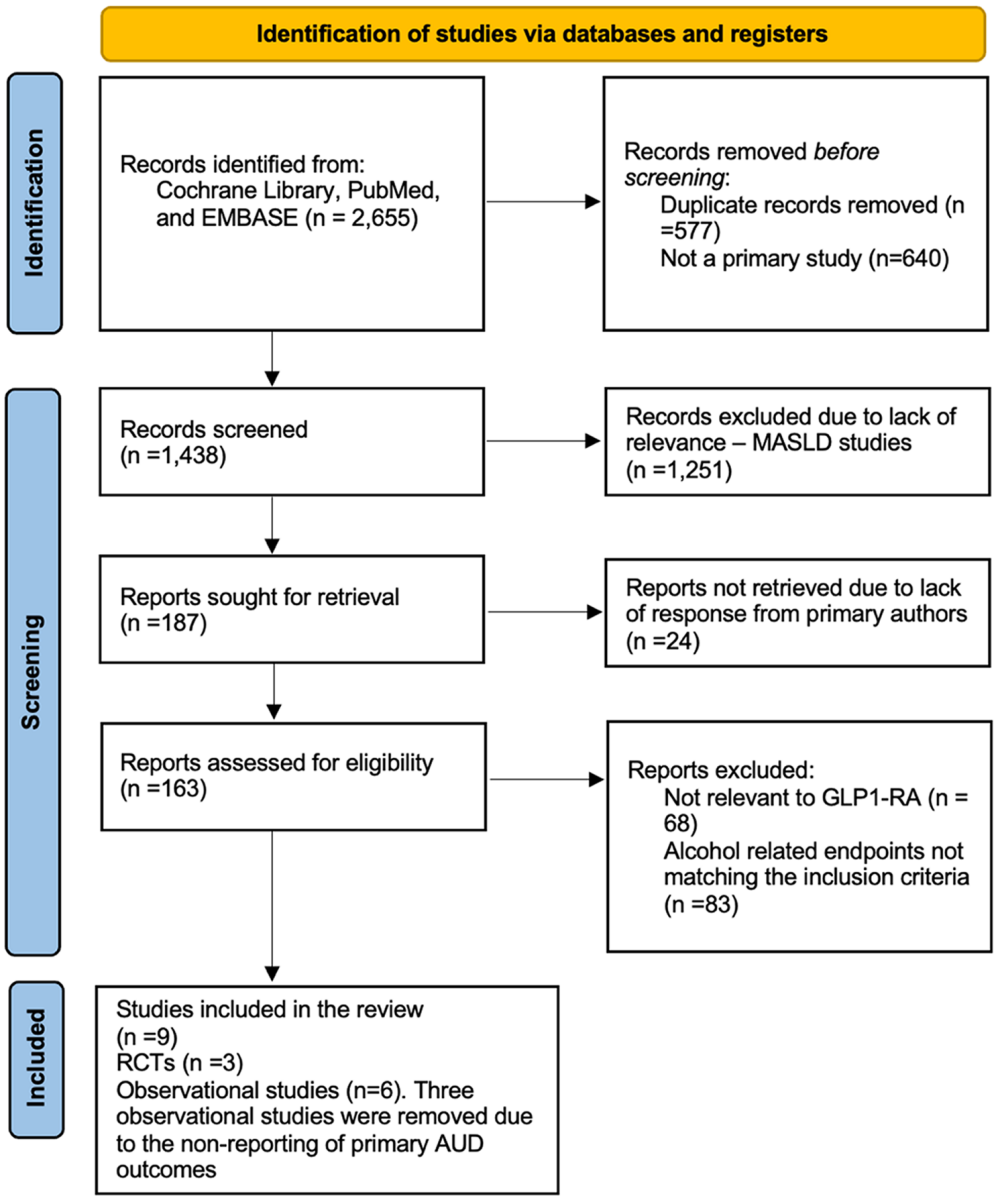
PRISMA Flow Diagram of Study Selection Process. The diagram illustrates the identification, screening, eligibility, and inclusion phases. Three studies were included in the systematic review but excluded from the meta-analysis due to ‘alcohol consumption not pooled,’ indicating incompatible data formats (e.g., median instead of mean) or insufficient reporting (e.g., missing standard deviations) for pooling, or case series design

Studies were eligible if they included adults (≥ 18 years) with or without diagnosed alcohol use disorder (AUD), including those with co-morbid obesity or type 2 diabetes mellitus (T2DM). Inclusion of adults with or without diagnosed AUD was intended to reflect real-world GLP-1RA use and assess both treatment and preventive effects, with subgroup analyses exploring population differences where feasible. The intervention was any GLP-1 RA-based therapy (e.g., semaglutide, liraglutide, exenatide, tirzepatide) compared to placebo, no treatment, or other pharmacological interventions (e.g., naltrexone, acamprosate, non-GLP-1 RA anti-obesity or anti-diabetes medications). Non-pharmacological interventions (e.g., behavioural therapies) were excluded as the primary comparator unless they were part of a control arm in a randomised controlled trial (RCT) or other controlled pharmacological study. Outcomes of interest for meta-analysis included alcohol consumption (total intake or drinks per drinking day, measured as standardised mean difference [SMD]), AUD incidence/recurrence (hazard ratios [HR] or odds ratios [OR]), heavy drinking days (OR), and alcohol craving (SMD, e.g., via Penn Alcohol Craving Scale). Secondary outcomes, such as quality of life related to alcohol use, weight change, and glycaemic control, were extracted but not pooled in this meta-analysis due to the insufficient data available. (Supplementary Table 2) Eligible study designs included randomised controlled trials (RCTs), cohort studies, case-control studies, and case series. Case series were included in the systematic review but excluded from the meta-analysis unless they provided suitable data for pooling (e.g., HR, SMD, OR). Observational studies required the use of propensity-score matching, within-individual designs, or similar robust methods (e.g., multivariable regression with adequate covariate adjustment) to control for confounding, thereby ensuring reliable estimates of effect. This criterion led to the exclusion of 24 studies during the full-text review due to inadequate control of confounders, as part of the 86 studies excluded for alcohol-related endpoints that did not meet the inclusion criteria, as detailed in the PRISMA flow diagram (Fig. 1). No studies using alternative robust methods, such as instrumental-variable approaches, were identified in the search, possibly due to the emerging nature of GLP-1RA research in AUD. Qualitative studies, case reports, cross-sectional studies without comparators, and observational studies without such confounder control methods were excluded. We acknowledge that this criterion may favour more recent studies that use advanced methods, such as propensity-score matching, which could potentially introduce selection bias.

### Search strategy

We conducted a comprehensive search of PubMed, Embase, and Cochrane Library (Trials) from January 1, 2005, to May 3, 2025, starting with the FDA approval of exenatide (April 28, 2005), the first GLP-1 receptor agonist. These databases were selected for their extensive coverage of biomedical, pharmacological, and clinical trial literature relevant to GLP-1 receptor agonists (GLP-1RAs) and alcohol-related outcomes. The search used Medical Subject Headings (MeSH) and free-text terms for alcohol-related outcomes (e.g., “Alcohol-Related Disorders“[MeSH], “alcohol use disorder,” “alcohol* consum*,” “alcohol craving,” “heavy drinking”) and GLP-1RAs (e.g., “Glucagon-Like Peptide 1“[MeSH], “semaglutide,” “liraglutide,” “exenatide,” “tirzepatide,” “albiglutide,” “dulaglutide,” “efpeglenatide”). An example PubMed search string was: (“Alcohol-Related Disorders“[MeSH Terms] OR “Alcohol Drinking“[MeSH Terms] OR “Alcoholism“[MeSH Terms] OR alcohol use disorder OR AUD OR alcohol* consum* OR alcohol craving OR heavy drinking) AND (“Glucagon-Like Peptide 1“[MeSH Terms] OR “Receptors, Glucagon“[MeSH Terms] OR GLP-1 receptor agonist* OR semaglutide OR liraglutide OR exenatide OR tirzepatide OR albiglutide OR dulaglutide OR efpeglenatide). Reference lists of included studies and trial registries (ClinicalTrials.gov, WHO ICTRP) were searched for ongoing and unpublished randomised controlled trials (RCTs). No unpublished RCTs meeting the inclusion criteria (e.g., assessing GLP-1RAs for alcohol-related outcomes in adults) were identified. No language restrictions were applied.

### Study selection

Records were deduplicated using EndNote software. Two reviewers (SG and BS) independently screened titles and abstracts using Covidence software, followed by full-text review, with discrepancies resolved by discussion. The selection process was documented in a PRISMA flow diagram (Fig. 1), which details the identification of 2,655 records from a database search (Cochrane Library, PubMed, and Embase) up to May 3, 2025. After removing 1,217 records before screening (577 duplicates, 640 non-primary studies), 1,438 records were screened, 187 reports were sought for retrieval, 24 reports were not retrieved due to lack of response from primary authors, 163 reports were assessed for eligibility, and nine studies were ultimately included in the meta-analysis (3 RCTs, six observational studies) after excluding 154 reports (68 not relevant to GLP1-RA, 83 alcohol-related endpoints not matching inclusion criteria). Additionally, three studies were included in the systematic review but excluded from the meta-analysis due to incompatible outcomes (alcohol consumption not pooled) or study design (case series), as detailed in Section “Baseline characteristics”.

### Data extraction

Two reviewers (SG and BS) independently extracted data using a standardised form, including study design, population characteristics (age, sex, BMI, AUD status), intervention details (type, dose, duration of GLP-1RA), comparator, outcomes, and risk of bias. Outcomes extracted included alcohol consumption (total intake or drinks per drinking day), with the latter reflecting country-specific standard-drink definitions (e.g., 14 g in the US, 12 g in Denmark); conversion to grams per day was not feasible due to inconsistent reporting, limiting direct volume comparability, AUD incidence/recurrence (as part of alcohol-related events in observational studies, not assessed in RCTs due to their short-term design focused on consumption and craving), alcohol craving, and heavy drinking days, defined as the mean number of days with ≥ 4 drinks for women or ≥ 5 drinks for men, with effects reported as standardized mean differences (SMDs). The data extraction form is provided in Supplementary Table 1 for transparency.

Secondary outcomes, such as weight change and glycaemic control, were also extracted but not pooled in the meta-analysis due to insufficient data. A summary of secondary outcome findings is provided in Supplementary Table 2. Authors were contacted for missing data (e.g., effect sizes, standard deviations).

### Risk of bias assessment

Two reviewers (SG and BS) independently assessed the risk of bias, with discrepancies resolved by consensus. RCTs were evaluated using the Cochrane Risk of Bias Tool 2 (RoB 2), and observational studies were assessed using the Newcastle-Ottawa Scale (NOS) [10, 11]. Case series were evaluated using a modified NOS for the systematic review, but were excluded from the meta-analysis due to study design. Publication bias was planned using funnel plots and Egger’s test for observational studies, while the trim-and-fill method was selected for RCTs [12]. Egger’s test was not planned to be conducted in case of small number of small number of studies (k < 10).

### Statistical analysis

Meta-analyses were conducted using the meta package in R (version 4.3.1). Continuous outcomes (e.g., alcohol consumption, craving) were pooled as standardized mean differences (SMD), and binary outcomes (e.g., alcohol-related events including AUD incidence/recurrence in observational studies, heavy drinking days in RCTs) were pooled as hazard ratios (HR) or odds ratios (OR) where sufficient data were available, using a random-effects model with Restricted Maximum Likelihood (REML) estimation and Hartung-Knapp (HK) adjustment to account for small numbers of studies.

Heavy drinking days were reported in one RCT but not pooled due to insufficient data. Heterogeneity was assessed using prediction intervals (PI), τ², and the Q test p-value, with sources explored through subgroup analyses (e.g., by GLP-1 RA type for RCTs, and by outcome type and GLP-1 RA type for observational studies).

Subgroup analyses were systematically conducted to investigate heterogeneity and effect modification, including: (a) GLP-1RA type (e.g., Semaglutide, Liraglutide, GIP/GLP-1RAs) for RCTs to assess craving reduction differences and for observational studies to evaluate outcome-specific effects, (b) outcome type (e.g., AUD incidence/recurrence vs. SUD hospitalization) for observational studies, and (c) baseline characteristics (e.g., AUD diagnosis, hazardous drinking status) in RCTs where data permitted, such as for craving subgroup analysis. These analyses employed random-effects models with REML and tested subgroup differences using the Q statistic, with significance set at *p* < 0.05.

Sensitivity analyses were performed using the DerSimonian-Laird (DL) method to compare with REML + HK, confirming the robustness of results across models (Supplementary Table S6). Additional sensitivity analyses excluded studies with a high risk of bias or small sample sizes (*n* < 15 per arm). For RCTs, high risk of bias was defined as ‘high’ or ‘some concerns’ in key domains of the Cochrane Risk of Bias Tool 2 (RoB 2), such as blinding or outcome measurement (Supplementary Table 3). For observational studies, high risk was defined as a Newcastle-Ottawa Scale (NOS) score less than 7. (Supplementary Table 4) Certainty of evidence was assessed using the GRADE approach [13]. (Supplementary Table 5)

## Result

### Baseline characteristics

Following the systematic search and screening process, nine studies were included in this meta-analysis, comprising three randomised controlled trials (RCTs) and six observational studies, as detailed in the PRISMA flow diagram (Fig. 1). The search identified 2,655 records from databases (Cochrane Library, PubMed, and Embase) up to May 3, 2025. After deduplication and exclusions, nine studies were included in the meta-analysis, encompassing 2,740,637 participants (430 from RCTs, 2,740,207 from observational studies). Additionally, three studies were included in the systematic review but excluded from the meta-analysis due to ‘alcohol consumption not pooled,’ reflecting incompatible data formats (e.g., median instead of mean) or insufficient reporting (e.g., missing standard deviations) for pooling, or study design (case series).

Baseline characteristics, including participant numbers per group, study country, and eligibility criteria, are summarized in Table 1. The RCTs included Hendershot et al. (2025) [14], Klausen et al. (2022) [15], and Probst et al. (2023) [16], all of which were conducted in outpatient settings with participants diagnosed with AUD. The overall risk of bias was low to moderate, with Hendershot et al. (2025) [14] showing moderate risk due to lack of blinding (open-label design), Klausen et al. (2022) [15] exhibiting moderate risk from potential missing outcome data, and Probst et al. (2023) [16] having moderate risk due to self-reported outcomes, as detailed in Supplementary Table 3.

The observational studies included Wium-Andersen et al. (2022) [17], Lähteenvuo et al. (2024) [18], Wang et al. (2024) [19], Qeadan et al. (2025) [20], Farokhnia et al. (2025) [21], and Xie et al. (2025) [22]. Most observational studies used propensity-score matching (PSM) or within-individual designs to assess alcohol-related outcomes in patients treated with GLP1-RAs, often in populations with co-morbid T2DM or obesity. The baseline characteristics of the studies included in the meta-analysis are summarised in Table 1. RCTs involved younger participants (mean age 42–48 years), with a higher proportion of males (60–70%), and focused on AUD-diagnosed individuals. Observational studies had a broader age range (mean age, 46–66 years) and included both AUD and non-AUD populations, often with comorbid conditions such as type 2 diabetes mellitus (T2DM) or obesity (mean BMI, 32–35 kg/m², where reported). Intervention durations varied, with RCTs ranging from 9 to 26 weeks and observational studies spanning 1 to 8.8 years (median). GLP1-RA-based therapies included semaglutide, liraglutide, dulaglutide, exenatide, and GIP/GLP1-RAs (e.g., tirzepatide), with comparators in RCTs being placebo and in observational studies often being non-GLP1-RA treatments, DPP-4 inhibitors, or no treatment. Outcomes assessed in the meta-analysis included alcohol consumption (SMD), alcohol craving (SMD), and heavy drinking days (OR) for RCTs, and alcohol-related events (HR/aHR) for observational studies.

Risk of bias assessments were conducted for all included studies (Supplementary Tables 3 and 4). For RCTs, the Cochrane Risk of Bias Tool 2 (RoB 2) indicated a low to moderate overall risk, with concerns primarily related to the blinding of participants due to the nature of the interventions. Observational studies, assessed using the Newcastle-Ottawa Scale (NOS), demonstrated good quality, with scores ranging from 7 to 8 out of 9; however, some studies had limitations in comparability due to residual confounding. The certainty of evidence was evaluated using the GRADE approach (Supplementary Table 5), with moderate certainty assigned to observational study outcomes and low certainty to RCT outcomes, due to heterogeneity and limited sample sizes.

**Table 1:** Baseline characteristics of included Studies, PSM: Propensity-score matched; NR: not reported. Effect type specifies the treatment effect analogue: “Initiation vs. Non-use” (intention-to-treat), “Initiation and sustaining vs. DPP-4i” (per-protocol), or “Receiving vs. No Treatment” (prevalent-user), based on study design

Study (Year, Country)	Study Design	Population (*N*)	Mean Age (Years)	% Male	Mean BMI (kg/m²)	Intervention (GLP1-RA)	Comparator	Duration	Eligibility Criteria	Effect Type
Hendershot et al. (2025, USA) [14]	RCT	AUD adults (48)	42	65	28	Semaglutide (*n* = 24)	Placebo (*n* = 24)	9 weeks	Adults ≥ 18 years with diagnosed AUD, no severe psychiatric disorders	N/A
Klausen et al. (2022, Denmark) [15]	RCT	AUD adults (127)	45	70	29	Exenatide (*n* = 62)	Placebo (*n* = 65)	26 weeks	Adults ≥ 18 years with AUD, no recent substance abuse	N/A
Probst et al. (2023, Switzerland) [16]	RCT	AUD adults (255)	48	60	27	Dulaglutide (*n* = 127)	Placebo (*n* = 128)	12 weeks	Adults ≥ 18 years with AUD, undergoing smoking cessation	N/A
Wium-Andersen et al. (2022, Denmark) [17]	Cohort (PSM)	T2DM adults (87,676)	58	55	32	Semaglutide/Liraglutide (*n* = 38,454)	DPP-4 inhibitors (*n* = 49,222)	4.1 years (median)	Adults ≥ 18 years with T2DM, no prior AUD diagnosis	Initiation and Sustaining vs. DPP-4i (Per-protocol)
Lähteenvuo et al. (2024, Sweden) [18]	Cohort (Within-individual)	AUD adults (227,868)	46	64	NR	Semaglutide/Liraglutide (*n* = 6,276)	Non-use periods (*n* = 221,592)	8.8 years (median)	Adults ≥ 18 years with diagnosed AUD	Initiation vs. Non-use (Intention-to-treat)
Wang et al. (2024, USA) [19]	Cohort (PSM)	Obese adults (83,825)	51	34	NR	Semaglutide (*n* = 45,797)	Non-GLP1-RA anti-obesity meds (*n* = 38,028)	1 year	Adults ≥ 18 years with obesity, no prior AUD diagnosis	Initiation vs. Non-use (Intention-to-treat)
Qeadan et al. (2025, USA) [20]	Cohort (Retrospective)	AUD adults (817,309)	53	49	NR	GIP/GLP1-RAs (*n* = 5,621)	No GIP/GLP1-RA (*n* = 811,688)	Up to 2 years	Adults ≥ 18 years with AUD or opioid use disorder	Receiving vs. No Treatment (Prevalent-user)
Farokhnia et al. (2025, UK & USA) [21]	Cohort (PSM)	AUD adults (54,462)	62.3	93.5	35.0	Semaglutide (*n* = 27,231)	No treatment (*n* = 27,231)	2 years	Adults ≥ 18 years with AUD, no severe comorbidities	Receiving vs. No Treatment (Prevalent-user)
Xie et al. (2025, USA) [22]	Cohort (PSM)	AUD adults (1,419,067)	65.96	92.38	35.02	GLP-1RAs (*n* = 215,970)	Usual care (non-GLP-1RA antihyperglycemics) (*n* = 1,203,097)	3.68 years (median)	Adults ≥ 18 years with AUD, T2DM, or obesity	Initiation vs. Usual Care (Intention-to-treat)

### Meta-analysis of RCTs

Three randomised controlled trials (RCTs)—Hendershot et al. (2025) [14], Klausen et al. (2022) [15], and Probst et al. (2023) [16]—involving 430 participants diagnosed with alcohol use disorder (AUD) were analysed to assess the effects of glucagon-like peptide-1 receptor agonists (GLP1-RAs) on alcohol-related outcomes. Outcomes included total alcohol consumption, drinks per drinking day, alcohol craving, and heavy drinking days, as defined in the short-term focus of Section “Data extraction”. A random-effects model with Hartung-Knapp (HK) adjustment was employed to account for the small number of studies and potential heterogeneity.

**Fig. 2:**
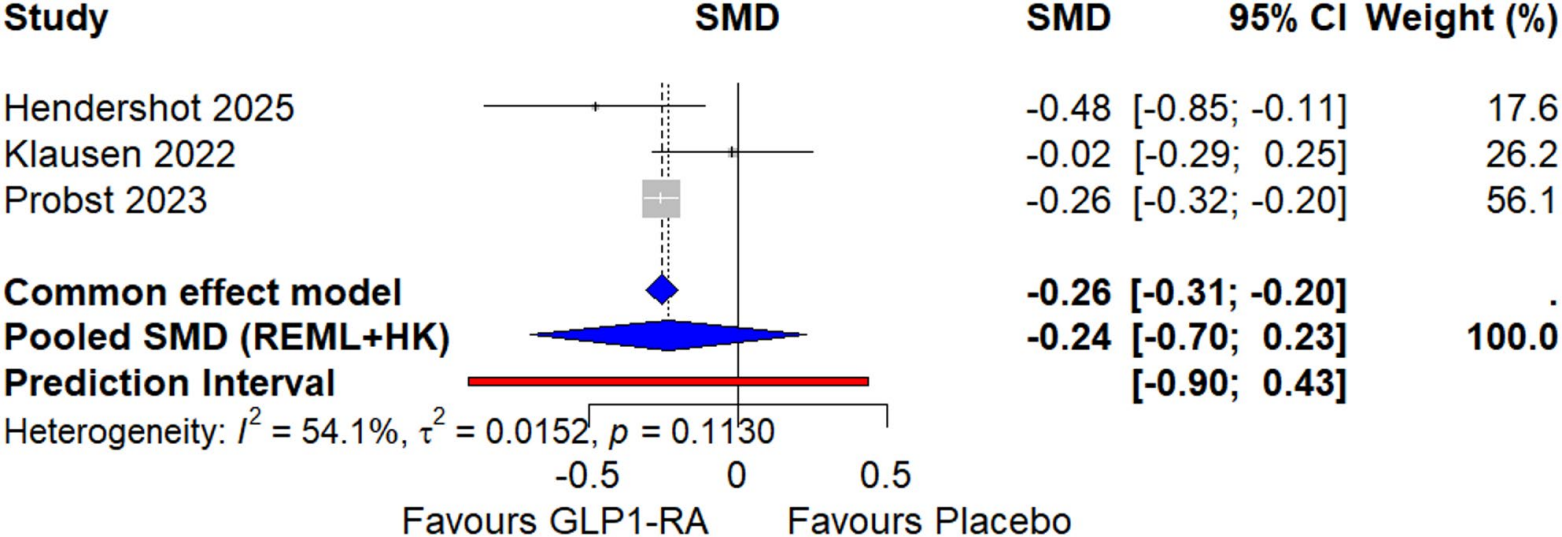
Forest plot of the meta-analysis of **randomised controlled trials** (RCTs) using a random-effects model with Restricted Maximum Likelihood (REML) estimation and Hartung-Knapp (HK) adjustment assessing the effect of **glucagon-like peptide-1 receptor agonists** (GLP-1RAs) on **total alcohol consumption**, measured as standardised mean differences (SMD). The plot includes three randomised controlled trials (RCTs): Hendershot et al. (2025; Semaglutide) [14], Klausen et al. (2022; Exenatide) [15], and Probst et al. (2023; Dulaglutide) [16]. The diamond represents the pooled SMD using a random-effects model with Hartung-Knapp (HK) adjustment, with a 95% confidence interval (CI). SMD values below zero favour GLP-1RAs over placebo

The overall effect for total alcohol consumption across the three RCTs is shown in Fig. 2. The pooled SMD was − 0.24 (95% CI: -0.70, 0.23), indicating a slight, non-significant reduction in alcohol consumption favouring GLP1-RAs (*p* = 0.159), as determined by a random-effects model with Restricted Maximum Likelihood (REML) estimation and the Hartung-Knapp (HK) adjustment.

Heterogeneity was present, as indicated by the prediction interval (PI) of -0.90 to 0.43, with 54.1% of the observed variance (I²) reflecting variation in true effects between studies (τ² = 0.015, *p* = 0.113). For drinks per drinking day, the pooled SMD was − 0.23 (95% CI: -0.64, 0.19), indicating a slight, non-significant reduction (*p* = 0.169), with moderate heterogeneity (prediction interval = -0.79 to 0.33). (Supplementary Figure S1) The SMD for drinks per drinking day is based on country-specific definitions (e.g., 14 g in the US, 12 g in Denmark), with conversion to grams per day not possible due to data limitations. For alcohol craving (PACS), data were available from Hendershot et al. (2025) [14] and Klausen et al. (2022) [15], with a pooled SMD of -0.14 (95% CI: -2.84, 2.55, *p* = 0.156) and high heterogeneity (PI = -4.57 to 4.28, I² = 82.9%, τ² = 0.0763, *p* = 0.015). (Supplementary Figure S2) Potential self-reported outcome bias, referring to measurement error or recall bias in outcomes (e.g., alcohol consumption, craving) collected via participant self-reports, which may be influenced by social desirability or inaccurate memory, as assessed under the ‘bias in measurement of the outcome’ domain of the Cochrane Risk of Bias Tool 2 (RoB 2) (Supplementary Table 3), was noted in all RCTs. For heavy drinking days, data were only available from Hendershot et al. (2025) [14], with an OR of 0.84 (95% CI: 0.71, 1.00), indicating a non-significant reduction. This outcome was not pooled in the meta-analysis due to insufficient data, meaning it was reported in only one RCT, preventing the estimation of a pooled effect.

#### Subgroup analysis of RCTs by GLP1-RA type

Subgroup analyses by type of GLP-1RA used (semaglutide, exenatide, dulaglutide) were conducted for total alcohol consumption, drinks per drinking day, and alcohol craving to explore potential differences in treatment effects (Supplementary Figures S3, S4, S5). Hendershot et al. (2025) [14] used semaglutide, Klausen et al. (2022) [15] used exenatide, and Probst et al. (2023) [16] used dulaglutide. Given that each subgroup contains only one study, the analysis presents the individual standardized mean differences (SMDs) reported in the respective trials (e.g., Hendershot: -0.48 and Klausen: -0.02 for total alcohol consumption; Hendershot: -0.48, Klausen: -0.02, Probst: -0.26 for drinks per drinking day; Hendershot: -0.39, Klausen: 0.01 for alcohol craving) without statistical pooling or comparison due to insufficient data for between-group testing. Semaglutide showed the most potent effect, defined as the largest negative SMDs (-0.48, -0.41, -0.39), compared to exenatide (0.01 to -0.02) and dulaglutide (-0.26). However, a statistical comparison was not feasible due to the single-study design per subgroup. This approach aligns with the original trial effect sizes, as no synthesis was possible within the subgroups. Due to the small number of RCTs (k = 2–3), these analyses have limited statistical power, and the results should be interpreted cautiously. Subgroup analyses combining RCTs and observational studies were not performed due to differing outcome measures (SMD for RCTs vs. HR for observational studies) and study designs, which could introduce heterogeneity and compromise interpretability.

For total alcohol consumption, the forest plot (Supplementary Figure S3) showed the following subgroup effects: semaglutide (SMD: -0.48, 95% CI: -0.85, -0.11), exenatide (SMD: -0.02, 95% CI: -0.29, 0.25), and dulaglutide (SMD: -0.26, 95% CI: -0.32, -0.20). The overall random-effects model, with Restricted Maximum Likelihood (REML) estimation and Hartung-Knapp (HK) adjustment, yielded an SMD of -0.24 (95% CI: -0.70, 0.23), with a prediction interval of -0.91 to 0.43 (I² = 54.1%, τ² = 0.015, *p* = 0.113). The subgroup differences test was insignificant (χ² = 4.36, df = 2, *p* = 0.113).

For drinks per drinking day, the forest plot (Supplementary Figure S4) indicated subgroup effects as follows: semaglutide (SMD: -0.41, 95% CI: -0.73, -0.09), exenatide (SMD: -0.02, 95% CI: -0.29, 0.25), and dulaglutide (SMD: -0.26, 95% CI: -0.40, -0.12). The overall random-effects model (HK) yielded an SMD of -0.23 (95% CI: -0.65, 0.20), with a prediction interval of -0.82 to 0.37 (I² = 46.0%, τ² = 0.010, *p* = 0.156). The subgroup differences test was insignificant (χ² = 3.70, df = 2, *p* = 0.156).

For alcohol craving, the forest plot (Supplementary Figure S5) included only two studies: semaglutide (SMD: -0.39, 95% CI: -0.73, -0.05) and exenatide (SMD: 0.01, 95% CI: -0.08, 0.10). The overall random-effects model (HK) yielded an SMD of -0.16 (95% CI: -2.66, 2.35), with a prediction interval of -4.24 to 3.92 (I² = 80.3%, τ² = 0.064, *p* = 0.024). The test for subgroup differences was significant (χ² = 5.09, df = 1, *p* = 0.024), suggesting that semaglutide may have a more substantial effect on reducing alcohol craving compared to exenatide. The pooled SMD for alcohol craving in the primary analysis (SMD: -0.14, 95% CI: -2.84, 2.55) differs slightly from the subgroup analysis (SMD: -0.16, 95% CI: -2.66, 2.35) due to stratification by GLP-1RA type (Supplementary Figure S5).

#### Sensitivity analysis of RCTs

Sensitivity analyses excluded studies with potential bias or small sample sizes (*n* < 15 per arm). All three RCTs had sufficient sample sizes, and the risk of bias was low to moderate (Supplementary Table 3). Excluding Probst et al. (2023) [16], which had the highest risk of bias due to self-reported outcome bias, did not significantly alter the pooled SMD for total alcohol consumption (SMD: -0.23, 95% CI: -3.15, 2.68, *p* = 0.496; I² = 74.2%, τ² = 0.078). (Supplementary Figure S6).

#### Funnel plot asymmetry analysis of RCTs

Funnel Plot Asymmetry Analysis of RCTs Publication bias was assessed using a contour-enhanced funnel plot (Supplementary Figure S7) for the three RCTs, with no studies added via the trim-and-fill method (k = 3), indicating no missing studies were imputed. Contours are labelled with dark zones representing non-significant results (*p* > 0.10) and lighter zones indicating statistically significant results (*p* < 0.05). The random-effects model with the Hartung-Knapp (HK) adjustment yielded an SMD of -0.234 (95% CI: -0.679 to 0.211, t = -2.26, *p* = 0.152), consistent with the primary analysis. Heterogeneity was evaluated using the prediction interval (-0.86 to 0.39), which spans both protective and non-protective effects, suggesting variability across studies. The I² statistic of 52.3% (95% CI: 0.0%, 86.3%) indicates that 52.3% of the variability is due to true differences in effect sizes, with the remaining due to sampling error, consistent with moderate heterogeneity. The Q-test (Q = 4.19, df = 2, *p* = 0.122) was non-significant, likely due to the low power resulting from the small sample size of only three studies. Visual inspection of the contour-enhanced funnel plot revealed slight asymmetry, potentially indicating minor publication bias; however, with only three studies, no definitive conclusion can be drawn, and Egger’s test was not conducted due to its limited reliability with k < 10. Further discussion of this limitation is provided in Section “Discussion”.

### Meta-analysis of observational studies

**Fig. 3:**
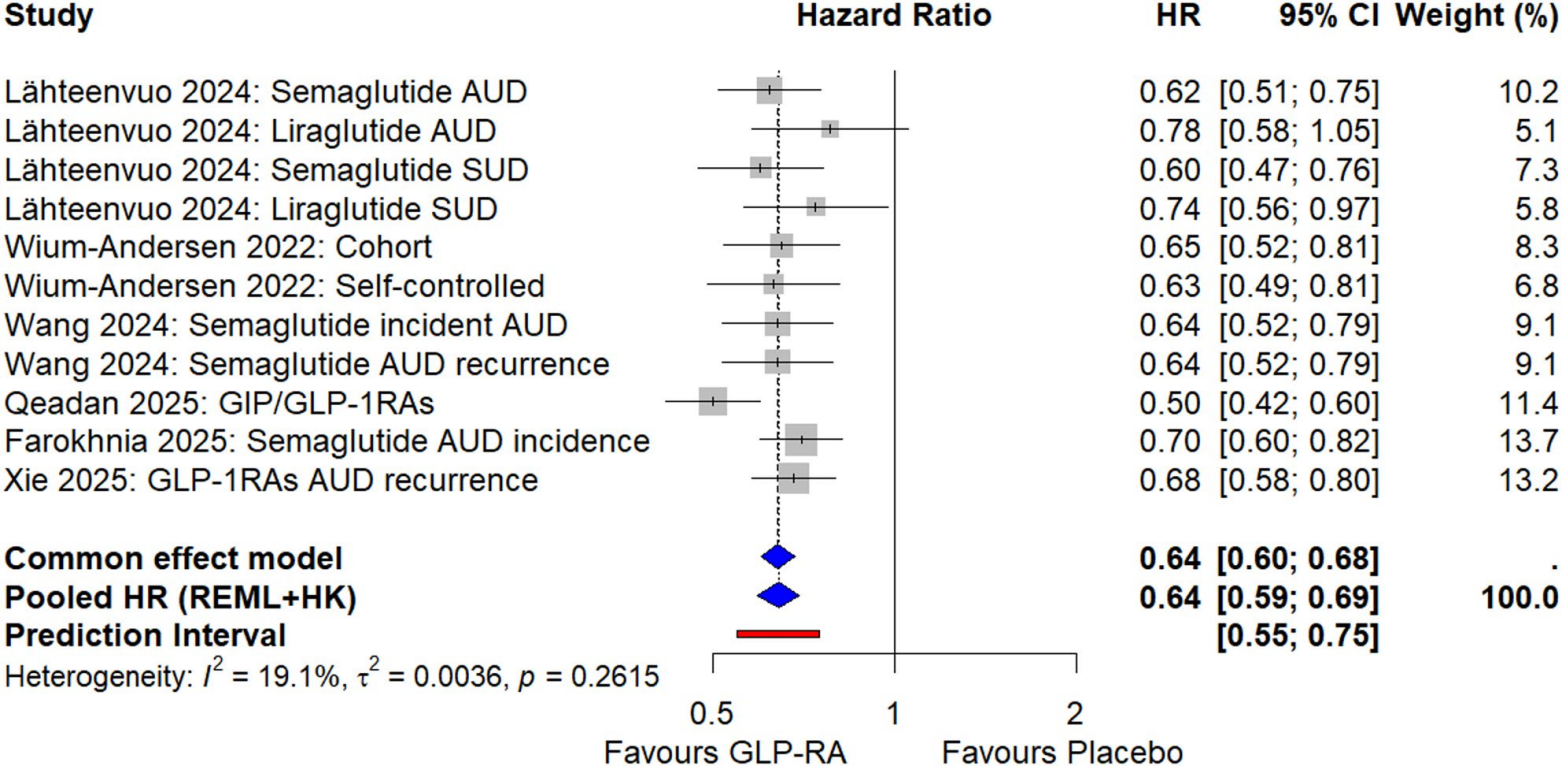
Forest plot of the meta-analysis of observational studies assessing the effect of glucagon-like peptide-1 receptor agonists (GLP1-RAs) on alcohol-related events, measured as hazard ratios (HR) or adjusted HR (aHR). The plot includes 11 estimates from six studies: Lähteenvuo et al. (2024; Semaglutide AUD, Liraglutide AUD, Semaglutide SUD, Liraglutide SUD) [18], Wium-Andersen et al. (2022; cohort and self-controlled designs) [17], Wang et al. (2024; Semaglutide incident AUD, Semaglutide AUD recurrence) [19], Qeadan et al. (2025; GIP/GLP1-RAs) [20], Farokhnia et al. (2025; Semaglutide AUD incidence) [21], and Xie et al. (2025; GLP-1RAs AUD recurrence) [22]. The diamond represents the pooled HR using a random-effects model with Hartung-Knapp (HK) adjustment, with a 95% confidence interval (CI). HR values below one favour GLP-1RAs over the control. Qeadan et al.’s intoxication outcome (HR = 0.50) is included in the pooled estimate but analysed narratively in Section “Alcohol intoxication outcome”

Six observational studies contributed 11 estimates assessing the effect of GLP-1RAs on alcohol-related events, which were pooled as hazard ratios (HRs) using a random-effects model with Restricted Maximum Likelihood (REML) estimation and the Hartung-Knapp (HK) adjustment. The pooled HR was 0.64 (95% CI: 0.59, 0.69, *p* < 0.001; I² = 19.1%, τ² = 0.003, *p* = 0.261), indicating that GLP-1RA use is associated with a statistically significant reduction in alcohol-related events. (Fig. 3) Alcohol-related events were defined as AUD incidence, AUD recurrence, SUD hospitalisation, or acute alcohol intoxication, with ‘AUD’ referring to incidence or recurrence unless specified (e.g., in subgroup analyses). In the included register-based study by Lähteenvuo et al., SUD hospitalisations were defined as a composite category encompassing both alcohol-related and non-alcohol-related substance use disorders; therefore, SUD hospitalisations inherently include alcohol-related admissions.

#### Subgroup analysis of observational studies

Subgroup analyses were conducted to explore clinical and methodological heterogeneity. A subgroup analysis by outcome type (Supplementary Figure S8) classified estimates into Alcohol Use Disorder (AUD) and substance use disorder (SUD) outcomes. For AUD outcomes (9 estimates from Lähteenvuo et al., 2024 [18]; Wium-Andersen et al., 2022 [17]; Wang et al., 2024 [19]; Qeadan et al., 2025 [20]; Farokhnia et al., 2025 [24]; Xie et al., 2025 [25];, the pooled HR was 0.64 (95% CI: 0.58, 0.70, I² = 28.6%, τ² = 0.005, *p* = 0.190), indicating a significant protective effect of GLP-1RAs on alcohol-specific outcomes. For SUD outcomes (2 estimates from Lähteenvuo et al., 2024 [18]; the pooled HR was 0.66 (95% CI: 0.17, 2.48; I² = 22.9%, τ² = 0.005, *p* = 0.254), indicating a non-significant effect. The difference between AUD and SUD subgroups was not significant (*p* = 0.793). Observational studies estimated impacts as follows: Lähteenvuo et al. (2024) [18] and Wang et al. (2024) [19] assessed initiating GLP-1RAs vs. non-use (intention-to-treat analogue), Wium-Andersen et al. (2022) [17] evaluated initiating and sustaining GLP-1RAs vs. DPP-4 inhibitors (per-protocol analogue), Qeadan et al. (2025) [20] and Farokhnia et al. (2025) [21] compared receiving GLP-1RAs vs. no treatment (prevalent-user comparison), and Xie et al. (2025) [22] assessed initiating GLP-1RAs vs. usual care (intention-to-treat analogue).

To further enhance specificity and address concerns about pooling heterogeneous outcomes (e.g., chronic AUD incidence/recurrence vs. acute alcohol intoxication), we conducted separate meta-analyses for AUD and SUD outcomes, reporting alcohol intoxication narratively (see Sect. “Funnel plot of observational studies”, “Separate meta-analyses for AUD and SUD outcomes” and “Subgroup analysis for separate meta-analyses”).

Subgroup analysis by GLP-1RA type (Supplementary Figure S9) revealed differences in effect sizes: Semaglutide (HR: 0.65, 95% CI: 0.60, 0.70, I² = 0; 5 estimates from Lähteenvuo et al., 2024 [18]; Wang et al., 2024 [19]; Farokhnia et al., 2025 [21]) showed a stronger protective effect compared to Liraglutide (HR: 0.76, 95% CI: 0.54, 1.06, I² = 0%; 2 estimates from Lähteenvuo et al., 2024 [18]), with a significant subgroup difference (*p* < 0.001). GIP/GLP-1RAs (Qeadan et al., 2025 [20]) demonstrated the most potent effect (HR: 0.50, 95% CI: 0.42, 0.60; 1 estimate), while studies with mixed GLP-1RAs (Wium-Andersen et al., 2022 [17]; Xie et al., 2025 [22]) had a pooled HR of 0.66 (95% CI: 0.60, 0.73, I² = 0%; 3 estimates). The ‘strongest effects’ are defined as the largest reductions in HR (GIP/GLP-1RAs: 0.50; Semaglutide: 0.65) and the most significant p-values (p < 0.0001 for both), compared to Liraglutide (0.76, p = 0.105) and Mixed GLP-1RAs (0.66, p < 0.0001). However, a statistical comparison between subgroups was not feasible due to each subgroup containing only one study per agent, except for Mixed GLP-1RAs, though the overall p-value for subgroup differences was < 0.0001, indicating significant heterogeneity.

#### Sensitivity analysis of observational studies

A sensitivity analysis was performed to assess the robustness of the findings by excluding studies with a high risk of bias (Supplementary Figure S10). Since all included studies scored ≥ 7 on the Newcastle-Ottawa Scale (Supplementary Table 4), no studies were excluded. The pooled HR remained unchanged from the primary analysis (HR: 0.64, 95% CI: 0.59, 0.69, *p* < 0.001; I² = 20.5%, τ² = 0.0039, *p* = 0.249), confirming the robustness of the findings across all 11 estimates from the six observational studies. Additionally, a sensitivity analysis using the DerSimonian-Laird (DL) method instead of Restricted Maximum Likelihood (REML) yielded consistent results (HR: 0.64, 95% CI: 0.59, 0.69, *p* < 0.001; I² = 19.1%, τ² = 0.0026, *p* = 0.262), further supporting the robustness of the findings to the choice of random-effects model (Supplementary Table 6).

#### Funnel plot of observational studies

Funnel Plot of Observational Studies Publication bias was assessed using a contour-enhanced funnel plot (Supplementary Figure S11) for the six observational studies contributing eleven estimates, with no studies added via the trim-and-fill method (k = 11 estimates), indicating no missing studies were imputed. Contours are labelled with dark zones representing non-significant results (*p* > 0.10) and lighter zones indicating statistically significant results (*p* < 0.05). Visual inspection of the contour-enhanced funnel plot, which displays all eleven estimates, revealed symmetry, indicating no evidence of publication bias. Additionally, an exploratory Egger’s test was conducted on the eleven estimates, showing no significant asymmetry (*p* = 0.555); however, this result should be interpreted with caution, as the test’s reliability is limited by the six independent studies (k = 6), which is below the recommended threshold of ten for robust application, consistent with the approach for RCTs.

#### Separate meta-analyses for AUD and SUD outcomes

**Fig. 4:**
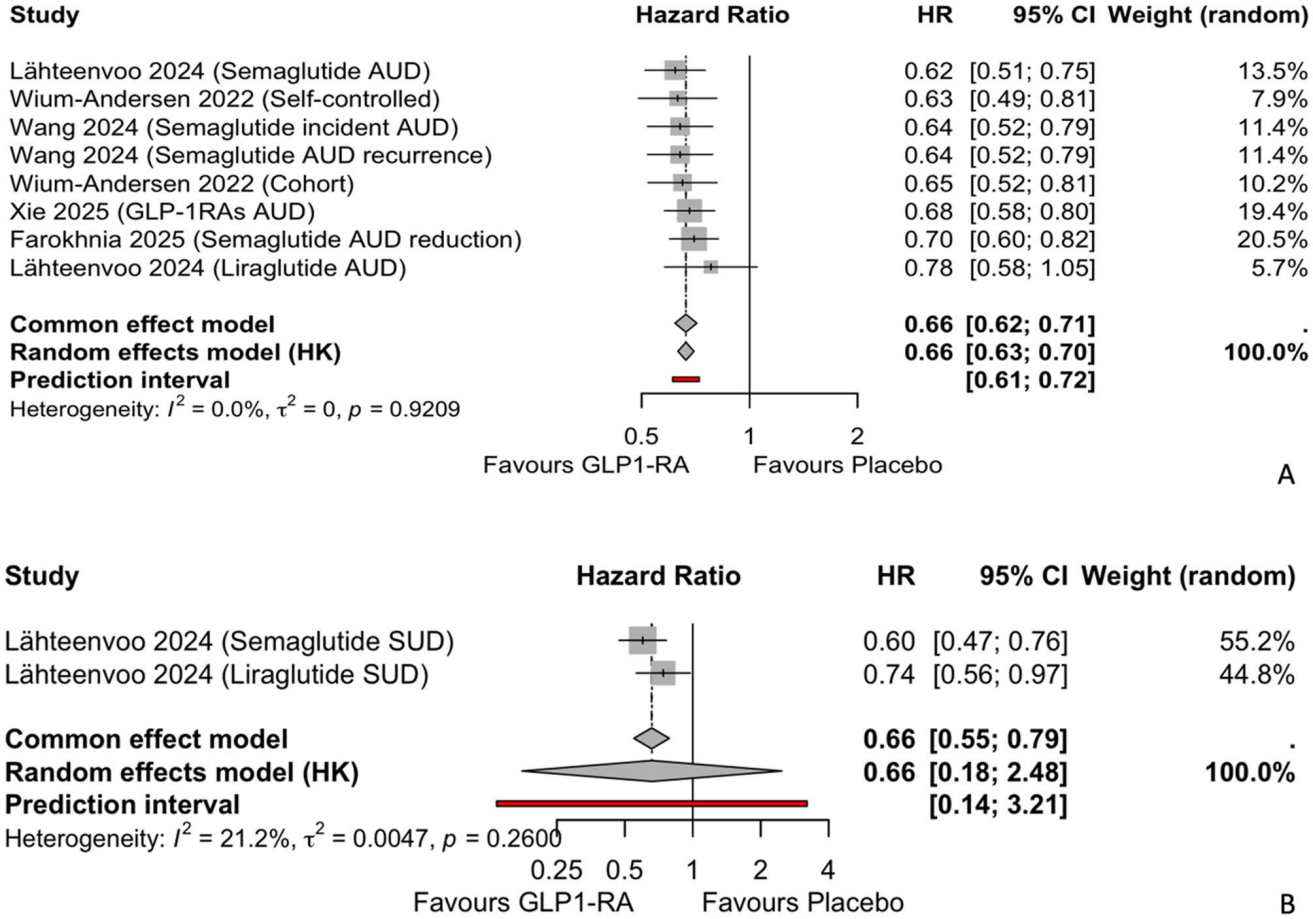
Forest plots of separate meta-analyses for (A) AUD outcomes (k = 8) and (B) SUD outcomes (k = 2), using random-effects models with REML and HK adjustment. HR values below one favour GLP-1RAs

To enhance specificity and interpretability, separate meta-analyses were conducted for AUD and SUD outcomes (Fig. 4), excluding the alcohol intoxication outcome from Qeadan et al. (2025) [20] to avoid pooling heterogeneous constructs (chronic vs. acute events).

The AUD meta-analysis (k = 8) yielded an HR of 0.66 (95% CI: 0.63–0.70, *p* < 0.0001), with no heterogeneity (PI = 0.61 to 0.72). The SUD meta-analysis (k = 2) yielded an HR of 0.66 (95% CI: 0.18–2.48, *p* = 0.156), with evidence of heterogeneity (PI = 0.14 to 3.21). However, the wide CI and non-significant p-value reflect limited statistical power.

#### Subgroup analysis for separate meta-analyses

Subgroup analyses by GLP-1RA type were performed for the separate AUD and SUD meta-analyses (Supplementary Figures S12 and S13). For AUD outcomes, no significant subgroup differences were found (*p* = 0.532), with Semaglutide (HR: 0.66, 95% CI: 0.60–0.72, k = 4, I² = 0.0%), Mixed GLP-1RAs (HR: 0.66, 95% CI: 0.60–0.73, k = 3, I² = 0.0%), and Liraglutide (HR: 0.78, 95% CI: 0.58–1.05, k = 1). For SUD outcomes, Semaglutide (HR: 0.60, k = 1) showed a more substantial effect than Liraglutide (HR: 0.74, k = 1), but statistical comparison was not feasible.

#### Alcohol intoxication outcome

Qeadan et al. (2025) [20] reported an HR of 0.50 (95% CI: 0.42–0.60) for alcohol intoxication with GIP/GLP-1RAs. A meta-analysis was not feasible due to the presence of only a single study. This finding suggests a potentially meaningful association with fewer acute alcohol-related events, but confirmation in additional studies is required. In the original AUD subgroup analysis (Supplementary Figure S8), this study was included; however, it is reported separately here to enhance the specificity of the outcomes.

#### Sensitivity and publication bias for separate analyses

Sensitivity analyses for AUD and SUD outcomes included all studies (NOS ≥ 7), confirming the robustness of the results. Funnel plots for AUD outcomes (Supplementary Figure S14) showed no asymmetry on visual inspection. Egger’s test was not conducted due to k < 10. SUD funnel plots (Supplementary Figure S15) were limited by k = 2.

## Discussion

This systematic review and meta-analysis explored the potential of glucagon-like peptide-1 receptor agonists (GLP-1RAs) in managing alcohol use disorder (AUD), synthesising data from three randomised controlled trials (RCTs) and six observational studies encompassing 2,740,637 participants. The findings indicate distinct patterns across study designs, with RCTs showing limited evidence for reducing alcohol consumption and craving. In contrast, observational studies show a consistent association between GLP-1RA use and lower rates of alcohol-related events, particularly for chronic AUD outcomes, although the observational design precludes firm causal inference.

RCTs (*N* = 430) showed non-significant trends for reduced alcohol consumption, drinks per drinking day, and craving, with Semaglutide demonstrating more potent effects on craving in subgroup analyses. These findings align with Subhani et al. (2024) [24], who noted no overall impact on heavy drinking days but suggested benefits in obese patients [24]. The limited statistical power and short durations (9–26 weeks) of RCTs may explain the lack of significance, underscoring the need for larger, longer-term trials to confirm the efficacy of GLP-1RAs in AUD populations.

Observational studies (*N* = 2,740,207) consistently demonstrated a protective effect against alcohol-related events, with separate analyses confirming benefits for AUD outcomes, and preliminary evidence for reduced acute intoxication events. These results align with those of Singal et al., who highlighted reduced drinking and hospitalisation in real-world settings, and Moraes et al., who reported more substantial effects for events, possibly due to broader outcome inclusion [23, 25]. Semaglutide and GIP/GLP-1RAs (e.g., Tirzepatide) showed more potent effects, potentially reflecting enhanced modulation of reward pathways, as supported by neuroimaging studies [26]. The observed reductions in alcohol-related events may suggest a protective effect; however, this interpretation is tempered by potential biases, including residual confounding from unmeasured variables (e.g., socioeconomic status, mental health comorbidities), despite the use of robust methods like propensity-score matching. The seemingly stronger association observed for SUD hospitalisations compared with the null findings for non-alcohol drug outcomes reflects differences in outcome definitions across studies. In Lähteenvuo et al., SUD hospitalisations include both alcohol-related and non-alcohol-related admissions, whereas the ‘other drug use’ subgroup excludes alcohol-related events. Because reductions in alcohol-related outcomes primarily drive the associations observed in our analyses, the signal detected for SUD hospitalisations likely reflects the alcohol-related component rather than an effect on non-alcohol drug use.

The clinical implications are promising, particularly for patients with AUD and co-morbid metabolic conditions like obesity or type 2 diabetes mellitus, where GLP-1RAs are already indicated. The separate AUD and SUD analyses enhance specificity, suggesting that GLP-1RAs may be particularly effective for managing chronic AUD, with potential for preventing acute events. However, while disulfiram has variable efficacy, evidence-based Medications for Alcohol Use Disorders (MAUDs) such as naltrexone and acamprosate, which are significantly underutilised despite their proven benefits, should remain the first-line treatment pending conclusive RCT evidence on GLP-1RAs. Compared to these options, the favourable safety profile of GLP-1RAs in metabolic diseases could make them a safer adjunctive option, pending RCT validation [26].

### Strengths and limitations

This review’s strength lies in its comprehensive inclusion of both RCTs and observational studies, providing a broad perspective on the effects of GLP1-RAs across diverse populations (*N* = 2,740,637). The use of random-effects models with Hartung-Knapp adjustment, detailed subgroup analyses, and funnel plot assessment enhances the robustness of the findings. The addition of separate AUD and SUD meta-analyses addresses concerns about pooling heterogeneous outcomes, improving causal interpretability and clinical relevance.

However, several limitations must be acknowledged. The small number of RCTs (*n* = 3) and their short durations (9–26 weeks) limit their power to detect significant effects, particularly in subgroup analyses by GLP-1RA type (Supplementary Figures S3, S4, S5), which involved only 2–3 studies, thereby reducing statistical power and interpretability. The SUD analysis (k = 2) is underpowered, and single-study data limit conclusions regarding intoxication. The subgroup analysis by GLP-1RA type reflects individual trial effect sizes without pooling, due to the single-study limitation per subgroup, which limits the ability to assess between-group differences. Observational studies, although larger, are still subject to residual confounding, despite the use of propensity-score matching (PSM) or within-individual designs in all six studies, as required by the eligibility criteria. Although these methods controlled for measured confounders (e.g., age, sex, BMI, diabetes status), unmeasured variables such as socioeconomic status, mental health comorbidities (e.g., depression, anxiety), or lifestyle factors (e.g., smoking, diet) may not have been fully accounted for, potentially influencing the observed significant decrease in alcohol-related events (HR: 0.64, 95% CI: 0.59, 0.69). The requirement for observational studies to use PSM or similar methods resulted in the exclusion of 24 studies (Fig. 1), which may have introduced selection bias by favouring more recent studies with advanced methodological designs. Publication bias for separate analyses was assessed via visual inspection of funnel plots (Supplementary Figures S14, S15), with no Egger’s test performed due to k < 10. Accordingly, the apparent protective associations from observational studies should be interpreted as hypothesis-generating rather than definitive evidence of a treatment effect.

Additionally, the assessment of publication bias for RCTs relied on a visual inspection of a funnel plot, which included only three studies (Supplementary Figure S7), thereby limiting the reliability of detecting asymmetry and necessitating a cautious interpretation. Publication bias for separate analyses was assessed via visual inspection of funnel plots (Supplementary Figures S14, S15), with no Egger’s test performed due to k < 10. Furthermore, the inability to conduct a statistical comparison of subgroup effects by GLP-1RA type (e.g., Semaglutide, GIP/GLP-1RAs) in observational studies, due to single-study subgroups, limits the robustness of the claim about ‘strongest effects.’ Similarly, the lack of statistical comparison for RCT subgroups by GLP-1RA type (e.g., Semaglutide) restricts the ability to confirm the ‘strongest effect’ claim beyond descriptive differences in SMDs. Secondary outcomes (e.g., weight change, glycaemic control) were extracted but not pooled due to insufficient data (Supplementary Table 2).

The high heterogeneity in the RCT meta-analysis for alcohol craving (PI = -4.57 to 4.28) and the low heterogeneity in observational studies (PI = 0.55 to 0.75) suggest variability in study populations, GLP-1 RA types, and outcome definitions.

### Future directions

Future research should prioritise larger, longer-term RCTs to confirm the effects of GLP1-RAs on alcohol consumption and craving, with a focus on specific GLP1-RA types like Semaglutide and Tirzepatide, which showed promising results in subgroup analyses. Mechanistic studies exploring the neurobiological effects of GLP1-RAs on alcohol reward pathways, such as dopamine signalling in the ventral tegmental area, could further elucidate their therapeutic potential [27]. Additionally, studies in AUD populations without co-morbid metabolic conditions are needed to isolate the direct effects of GLP1-RAs on alcohol-related behaviours. Future research should focus on larger RCTs to validate GLP-1RA efficacy in AUD and explore the mechanisms underlying their effects. Ongoing trials (Supplementary Table 7) will provide further RCT evidence to confirm these findings and address current limitations [28–37]. Current evidence, primarily from observational studies, does not yet support guideline recommendations, which should await robust RCT data.

### Comparison with existing literature

The observational findings of this review, demonstrating a significant reduction in alcohol-related events (HR: 0.64), are consistent with those reported by Singal et al. (2025) [23], Subhani et al. (2024) [24], and Moraes et al. (2025) [25], which highlight GLP-1RAs’ potential in real-world settings. However, methodological differences distinguish these studies. Subhani et al.’s systematic review, which relies on narrative synthesis due to the limited availability of RCT data, aligns with our non-significant RCT trends (*p* = 0.159). However, their focus on consumption outcomes (e.g., heavy drinking days) contrasts with our event-based approach. Moraes et al.’s meta-analysis, pooling eight studies, reports a stronger HR (0.56, I² = 63.6%) for events, potentially reflecting their inclusion of liver-specific endpoints and higher heterogeneity, compared to our lower PI. Singal et al.’s commentary, citing observational and RCT evidence, underscores the dual benefit of AUD-ALD.

### Implications for policy and practice

The potential of GLP1-RAs to reduce alcohol-related events warrants consideration in clinical guidelines, particularly for patients with AUD and co-morbid metabolic conditions. However, policymakers should balance the promising observational data with the need for RCT confirmation, ensuring that any adoption of GLP1-RAs for AUD is supported by robust safety and efficacy data [38]. The separate AUD and SUD analyses suggest GLP-1RAs may be particularly effective for chronic AUD outcomes, guiding targeted use in practice.

## Conclusion

This systematic review and meta-analysis of nine studies indicates that, in observational cohorts, GLP-1RA use is associated with significantly lower rates of alcohol-related events, with some differences observed across GLP-1RA types. Semaglutide and GIP/GLP-1RAs (e.g., tirzepatide) showed numerically larger risk reductions than other agents, although these comparisons are based on limited, heterogeneous data. Separate analyses showed similar protective effects for AUD and SUD, with no heterogeneity in AUD outcomes. Preliminary evidence for alcohol intoxication suggests potential for acute event reduction. However, RCTs showed non-significant decreases in alcohol consumption, drinks per drinking day, and alcohol craving, with a significant subgroup difference favouring Semaglutide for craving reduction. These findings suggest that GLP-1RAs may hold promise as a novel therapeutic option for AUD, particularly in patients with comorbid metabolic disease; however, larger, longer-term RCTs are essential before GLP-1RAs can be recommended for this indication in routine practice.

## Supplementary Information

Below is the link to the electronic supplementary material.

Supplementary Material 1

## Data Availability

Available upon reasonable request.
