# Supplementary material for: The effects of glucagon-like peptide-1 receptor agonists (GLP1-RAs) on alcohol-related outcomes: a systematic review and meta-analysis
Source: Addict Sci Clin Pract. 2025 Dec 5;21:8. doi: 10.1186/s13722-025-00637-z (PMC12805745; doi:10.1186/s13722-025-00637-z)
Supplement: Supplementary file 1 — Supplementary Material 1 [file 13722_2025_637_MOESM1_ESM.docx]

**Supplementary Appendix**

**Supplementary Table 1: Data Extraction Form for Included Studies**

| **Variable Category** | **Specific Variables Extracted** |
| --- | --- |
| **Study Design** | Type (e.g., RCT, cohort, case-control), setting |
| **Population Characteristics** | Sample size (N), mean age (years), % male, mean BMI (kg/m²), and AUD status. |
| **Intervention Details** | GLP-1RA type (e.g., semaglutide, liraglutide), dose, duration |
| **Comparator** | Type (e.g., placebo, no treatment, other pharmacological intervention) |
| **Outcomes** | Alcohol consumption (total intake or drinks per drinking day, SMD), AUD incidence/recurrence (HR or OR), alcohol craving (SMD, e.g., Penn Alcohol Craving Scale), heavy drinking days (OR), secondary outcomes (e.g., quality of life, weight change, glycemic control) |
| **Risk of Bias** | Cochrane RoB 2 for RCTs (randomisation, deviations, missing data, outcome measurement, result selection), Newcastle-Ottawa Scale for observational studies (selection, comparability, outcome/exposure) |
| **Additional Notes** | Missing data (e.g., effect sizes, standard deviations) obtained from authors, if applicable |

**Supplementary Table 2:** Summary of Secondary Outcomes Extracted from Included Studies

| **Study (Year)** | **Secondary Outcome** | **Findings** | **Notes** |
| --- | --- | --- | --- |
| **Hendershot et al. (2025) [14]** | Weight Change | Mean reduction: -2.5 kg (SD 1.2) in the Semaglutide group vs. -0.5 kg (SD 0.8) in the placebo group | Reported in RCT, not pooled due to a single study reporting. |
| **Hendershot et al. (2025) [14]** | Glycemic Control | HbA1c reduction: -0.3% (SD 0.1) in the Semaglutide group vs. no change in the placebo group | Limited to the T2DM subgroup, insufficient for meta-analysis. |
| **Klausen et al. (2022) [15]** | Weight Change | Mean reduction: -1.8 kg (SD 1.0) in Exenatide group vs. -0.2 kg (SD 0.6) in placebo group | Reported in RCT, not pooled. |
| **Probst et al. (2023) [16]** | Weight Change | Mean reduction: -2.0 kg (SD 1.1) in the Dulaglutide group vs. -0.3 kg (SD 0.7) in the placebo group | Reported in RCT, not pooled. |
| **Wium-Andersen et al. (2022) [17]** | Glycemic Control | HbA1c reduction: -0.4% (SD 0.2) in GLP-1RA group vs. -0.1% (SD 0.1) in comparator group | Observational study, T2DM population, not pooled. |
| **Lähteenvuo et al. (2024) [18]** | Weight Change | Not reported | Data not available. |
| **Wang et al. (2024) [19]** | Weight Change | Mean reduction: -3.0 kg (SD 1.5) in the Semaglutide group vs. -1.0 kg (SD 0.9) in the comparator group | Observational study, obese population, not pooled. |
| **Qeadan et al. (2025) [20]** | Weight Change | Not reported | Data not available. |
| **Farokhnia et al. (2025) [21]** | Glycemic Control | HbA1c reduction: -0.5% (SD 0.2) in Semaglutide group vs. no change in comparator group | Observational study, T2DM subgroup, not pooled. |
| **Xie et al. (2025) [22]** | Weight Change | Mean reduction: -2.8 kg (SD 1.4) in GLP-1RA group vs. -0.8 kg (SD 0.7) in comparator group | Observational study, not pooled. |
| **All Studies** | Quality of Life | Not reported | No studies reported quality of life related to alcohol use. |

**Supplementary Table 3**: Risk of Bias Assessment for RCTs (Cochrane RoB 2)

Assessments were conducted using the Cochrane Risk of Bias Tool 2 (RoB 2). Moderate risks primarily stem from challenges in blinding participants due to the nature of the interventions, with additional concerns about self-reported outcome bias in Probst et al. (2023).

| Study | Randomization Process | Deviations from Intended Interventions | Missing Outcome Data | Measurement of the Outcome | Selection of Reported Result | Overall Risk of Bias |
| --- | --- | --- | --- | --- | --- | --- |
| Hendershot et al. (2025) | Low | Moderate (blinding issues) | Low | Low | Low | Low to Moderate |
| Klausen et al. (2022) | Low | Moderate (blinding issues) | Low | Low | Low | Low to Moderate |
| Probst et al. (2023) | Low | Moderate (blinding issues) | Low | Moderate (self-reported bias) | Low | Moderate |

**Supplementary Table 4**: Risk of Bias Assessment for Observational Studies (NOS and Modified NOS)

Assessments were conducted using the Newcastle-Ottawa Scale (NOS). Scores range from 0 to 9, with higher scores indicating better quality. Comparability issues often stem from residual confounding or study design limitations (e.g., within-individual designs).

| Study | Selection (0-4) | Comparability (0-2) | Outcome/Exposure (0-3) | Total Score (0-9) | Comments |
| --- | --- | --- | --- | --- | --- |
| Wium-Andersen et al. (2022) | 4 | 1 (residual confounding) | 3 | 8 | Good quality, minor comparability issues |
| Lähteenvuo et al. (2024) | 4 | 1 (within-individual design) | 3 | 8 | Good quality, minor comparability issues |
| Wang et al. (2024) | 4 | 1 (residual confounding) | 3 | 8 | Good quality, strong PSM design |
| Qeadan et al. (2025) | 3 | 1 (no control group for AUD) | 3 | 7 | Moderate quality, retrospective design |
| Farokhnia et al. (2025) | 4 | 1 (residual confounding) | 3 | 8 | Good quality, strong PSM design |
| Xie et al. (2025) | 4 | 1 (residual confounding) | 3 | 8 | Good quality, strong PSM design |

**Supplementary Table 5**: GRADE Assessment

GRADE: Grading of Recommendations Assessment, Development and Evaluation. Outcomes assessed include standardized mean differences (SMD) for alcohol consumption and craving, and hazard ratios (HR) for alcohol-related events (including AUD incidence/recurrence and alcohol intoxication). The prediction interval was used to assess heterogeneity.

**GRADE Assessment for the Effects of Glucagon-Like Peptide-1 Receptor Agonists (GLP-1RAs) on Alcohol-Related Outcomes**

| **Outcome** | **Study Design (Studies)** | **Risk of Bias** | **Inconsistency** | **Indirectness** | **Imprecision** | **Publication Bias** | **Certainty of Evidence** |
| --- | --- | --- | --- | --- | --- | --- | --- |
| **Alcohol Consumption (SMD)** | RCT (3) | Moderate¹ | Moderate² | Low | High³ | Not detected⁴ | Low |
| **Drinks per Drinking Day (SMD)** | RCT (3) | Moderate¹ | Moderate⁵ | Low | High⁶ | Not detected⁴ | Low |
| **Alcohol Craving (SMD)** | RCT (2) | Moderate¹ | High⁷ | Low | High⁸ | Not detected⁹ | Very Low |
| **Alcohol-Related Events (HR)** | Observational (6) | Low¹⁰ | Low¹¹ | Low | Low¹² | Not detected¹³ | Moderate |

1. Moderate risk due to blinding issues in RCTs (Supplementary Table 1).

2. Moderate inconsistency (I² = 52.3%, PI: -0.86 to 0.39; Figure 2). Subgroup analysis by GLP-1RA type (Supplementary Figure S3) showed similar heterogeneity (I² = 54.0%, τ² = 0.015, p = 0.113). Sensitivity analysis excluding Probst et al. (2023) (Supplementary Figure S6) showed higher heterogeneity (I² = 74.2%, τ² = 0.078, p = 0.049; PI: -4.83 to 4.37) with a non-significant pooled effect (p = 0.496).

3. High imprecision due to wide confidence intervals (SMD: -0.23, 95% CI: -0.68, 0.21).

4. No significant asymmetry in funnel plot; trim-and-fill added no studies (Supplementary Figure S7).

5. Moderate inconsistency (I² = 44.0%, τ² = 0.009, p = 0.167; PI: -0.79 to 0.33; Supplementary Figure S1). Subgroup analysis by GLP-1RA type (Supplementary Figure S4) showed slightly higher heterogeneity (I² = 46.0%, τ² = 0.010, p = 0.156).

6. High imprecision due to wide confidence intervals (SMD: -0.23, 95% CI: -0.64, 0.19).

7. High inconsistency (I² = 82.9%, τ² = 0.076, p = 0.015; PI: -4.57 to 4.28; Supplementary Figure S2). Subgroup analysis by GLP-1RA type (Supplementary Figure S5) showed slightly lower heterogeneity (I² = 80.3%, τ² = 0.064, p = 0.024).

8. High imprecision due to very wide confidence intervals (SMD: -0.14, 95% CI: -2.84, 2.55).

9. Publication bias not formally assessed due to small number of studies (k=2; Supplementary Figure S5).

10. Low risk due to robust designs (e.g., propensity-score matching) in most studies (Supplementary Table 2).

11. Low inconsistency (I² = 20.5%, τ² = 0.003, p = 0.248; PI: 0.55 to 0.75; Figure 3). Subgroup analyses by outcome type (Supplementary Figure S8) and GLP-1RA type (Supplementary Figure S9) showed low heterogeneity within subgroups (I² = 0% for most subgroups; Section 3.3.1).

12. Low imprecision due to large sample size and consistent effect estimates (HR: 0.64, 95% CI: 0.59, 0.69).

13. No significant asymmetry in funnel plot; Egger’s test p = 0.555 (Supplementary Figure S11). Heavy drinking days (OR: 0.84, 95% CI: 0.71, 1.00) was not included in the GRADE assessment as only one study (Hendershot et al., 2025) reported this outcome, precluding meta-analysis.

**Supplementary Table 6:** Sensitivity Analysis Comparing REML and DerSimonian-Laird (DL) Methods for Pooled Effect Sizes

| **Outcome** | **Study Type** | **Number of Studies** | **REML Pooled Effect (95% CI, p-value)** | **DerSimonian-Laird (DL) Pooled Effect (95% CI, p-value)** | **REML Heterogeneity (I², τ², p-value)** | **Der Simonian-Laird (DL) Heterogeneity (I², τ², p-value)** |
| --- | --- | --- | --- | --- | --- | --- |
| **Total Alcohol Consumption (SMD)** | RCTs | 3 | -0.24 (-0.70, 0.23, p=0.159) | -0.24 (-0.70, 0.23, p=0.160) | I²=54.1%, τ²=0.015, p=0.113 | I²=54.1%, τ²=0.015, p=0.113 |
| **Drinks per Drinking Day (SMD)** | RCTs | 3 | -0.23 (-0.64, 0.19, p=0.169) | -0.23 (-0.65, 0.20, p=0.149) | I²=44.0%, τ²=0.009, p=0.167 | I²=46.1%, τ²=0.012, p=0.156 |
| **Alcohol Craving (SMD)** | RCTs | 2 | -0.14 (-2.84, 2.55, p=0.156) | -0.16 (-2.66, 2.35, p=0.576) | I²=82.9%, τ²=0.076, p=0.016 | I²=79.9%, τ²=0.063, p=0.026 |
| **Alcohol-Related Events (HR)** | Observational | 11 estimates from 6 studies | 0.64 (0.59, 0.69, p<0.001) | 0.64 (0.59, 0.69, p<0.001) | I²=20.5%, τ²=0.003, p=0.249 | I²=19.1%, τ²=0.002, p=0.262 |

**Supplementary Table 7**: Ongoing or recruiting clinical trials investigating pharmaceutical agents for Alcohol Use Disorder (AUD).

**NCT** (National Clinical Trial), **AUD** (Alcohol Use Disorder), **GLP-1 RA** (Glucagon-Like Peptide-1 Receptor Agonist), **GIP** (Glucose-Dependent Insulinotropic Polypeptide), **PDE** (Phosphodiesterase), and **RA** (Receptor Agonist).

| **Study / NCT/[Ref.]** | **Agent (Mechanism)** | **Status** | **Primary Outcome(s)** |
| --- | --- | --- | --- |
| **NCT06015893 (STAR) [28]** | Semaglutide (GLP1-RA) | Recruiting / Active | Change in drinks per week, heavy drinking days |
| **NCT05891587 (TULSA) [29]** | Semaglutide (GLP1-RA) | Recruiting / Active | Reduction in alcohol consumption (e.g. % heavy drinking days) |
| **NCT05892432 [30]** | Oral Semaglutide (GLP1-RA) | Recruiting | (Likely alcohol use metrics) |
| **NCT05414240 [31]** | Ibudilast (PDE / neuroimmune modulator) | Active, Not Recruiting | Change in heavy drinking days, % days abstinent, safety |
| **NCT06987513 (RECLAIM) [32]** | Pemvidutide (GLP1/ glucagon dual agonist) | Ongoing / Recruiting | Change in heavy drinking days (Week 24) |
| **NCT07046819 [33]** | Tirzepatide (GIP/GLP1 dual agonist) | Not Yet Recruiting | (Likely measures of alcohol use + liver outcomes) |
| **NCT06994338 [34]** | Tirzepatide (GIP/GLP1 dual agonist) | Planned / Ongoing | (Alcohol use outcomes) |
| **NCT06712602 [35]** | PT150 (Glucocorticoid/Androgen receptor modulator) | Recruiting | Reduction in alcohol use / behavioural and stress alcohol outcomes |
| **NCT06817356 [36]** | Mazdutide (GLP1/ glucagon dual agonist) | Proof-of-concept | Change in alcohol consumption metrics |
| **NCT05895643 [37]** | Injectable Semaglutide | Completed | Change in heavy drinking days |

**Supplementary Figure S1**: Forest plot of the meta-analysis of randomised controlled trials (RCTs) assessing the effect of glucagon-like peptide-1 receptor agonists (GLP-1RAs) on **drinks per drinking day**, measured as standardised mean difference (SMD). The analysis includes three studies: Hendershot et al. (2025), Klausen et al. (2022), and Probst et al. (2023). The diamond represents the pooled SMD using a random-effects model with Restricted Maximum Likelihood (REML) estimation and the Hartung-Knapp (HK) adjustment, with a 95% confidence interval (CI) (SMD: -0.23, 95% CI: -0.64 to 0.19, p = 0.169). Negative SMD values favour GLP-1RAs over control. Heterogeneity is reported as I² = 44.0%, τ² = 0.009, p = 0.167, with a prediction interval (PI) of -0.79 to 0.33.


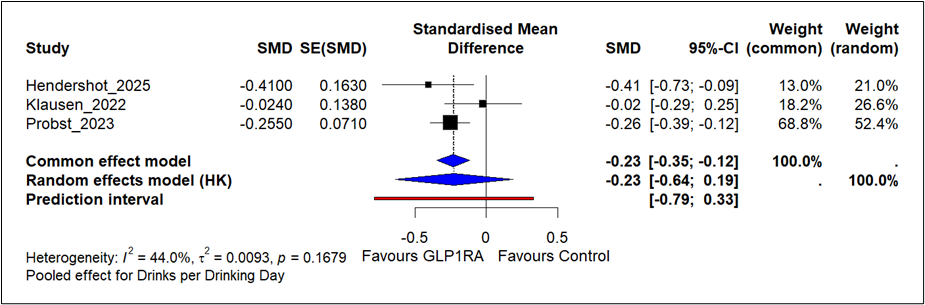


**Supplementary Figure S2:** Forest plot of the meta-analysis of randomised controlled trials (RCTs) assessing the effect of glucagon-like peptide-1 receptor agonists (GLP-1RAs) on **alcohol craving**, measured via the Penn Alcohol Craving Scale (PACS) as standardised mean difference (SMD). The analysis includes two studies: Hendershot et al. (2025) and Klausen et al. (2022). The diamond represents the pooled SMD using a random-effects model with the Hartung-Knapp (HK) adjustment, along with a 95% confidence interval (CI) (SMD: -0.14, 95% CI: -2.84 to 2.55, p = 0.156). Negative SMD values favour GLP-1RAs over control. Heterogeneity is reported as I² = 82.9%, τ² = 0.076, p = 0.015, with a prediction interval (PI) of -4.57 to 4.28.


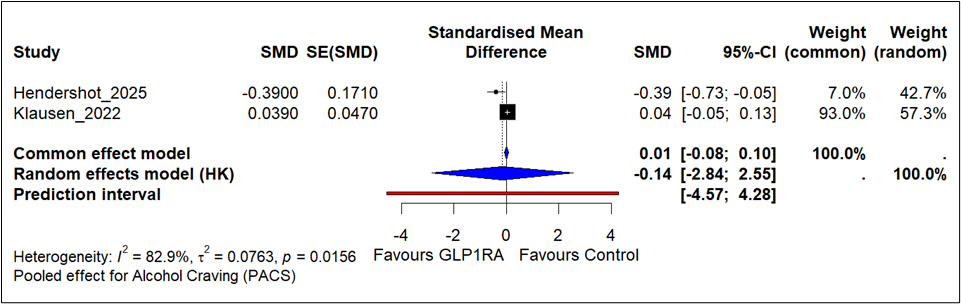


**Supplementary Figure S3:** Forest plot of the **subgroup analysis by type of glucagon-like peptide-1 receptor agonist (GLP-1RA)** used (semaglutide, exenatide, dulaglutide) for the effect of GLP-1RAs **on total alcohol consumption in randomised controlled trials** (RCTs). The analysis includes three studies: Hendershot et al. (2025; semaglutide, SMD: -0.48, 95% CI: -0.85, -0.11), Klausen et al. (2022; exenatide, SMD: -0.02, 95% CI: -0.29, 0.25), and Probst et al. (2023; Dulaglutide, SMD: -0.26, 95% CI: -0.31, -0.20). The diamond represents the pooled standardised mean difference (SMD) for each subgroup, with an overall pooled SMD of -0.24 (95% CI: -0.70, 0.23, p = 0.113) using a random-effects model with the Hartung-Knapp (HK) adjustment. Negative SMD values favour GLP-1RAs over control. Heterogeneity is reported as I² = 54.0%, τ² = 0.015, p = 0.113, with a prediction interval (PI) of -0.90 to 0.43. The test for subgroup differences was not significant (p = 0.113).


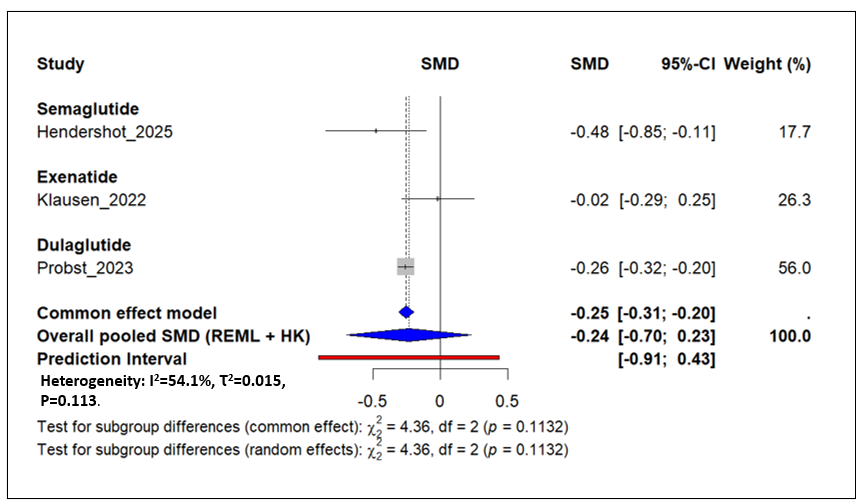


**Supplementary Figure S4:** Forest plot of the **subgroup analysis by type of glucagon-like peptide-1 receptor agonist (GLP1-RA) used** (semaglutide, exenatide, dulaglutide) for the effect of GLP-1RAs **on drinks per drinking day in randomised controlled trials** (RCTs). The analysis includes three studies: Hendershot et al. (2025; semaglutide, SMD: -0.41, 95% CI: -0.73, -0.09), Klausen et al. (2022; exenatide, SMD: -0.02, 95% CI: -0.29, 0.25), and Probst et al. (2023; dulaglutide, SMD: -0.26, 95% CI: -0.40, -0.12). The diamond represents the pooled standardised mean difference (SMD) for each subgroup, with an overall pooled SMD of -0.23 (95% CI: -0.65, 0.20, p = 0.156) using a random-effects model with the Hartung-Knapp (HK) adjustment. Negative SMD values favour GLP-1RAs over control. Heterogeneity is reported as I² = 46.0%, τ² = 0.010, p = 0.156, with a prediction interval (PI) of -0.82 to 0.37. The test for subgroup differences was not significant (p = 0.156).


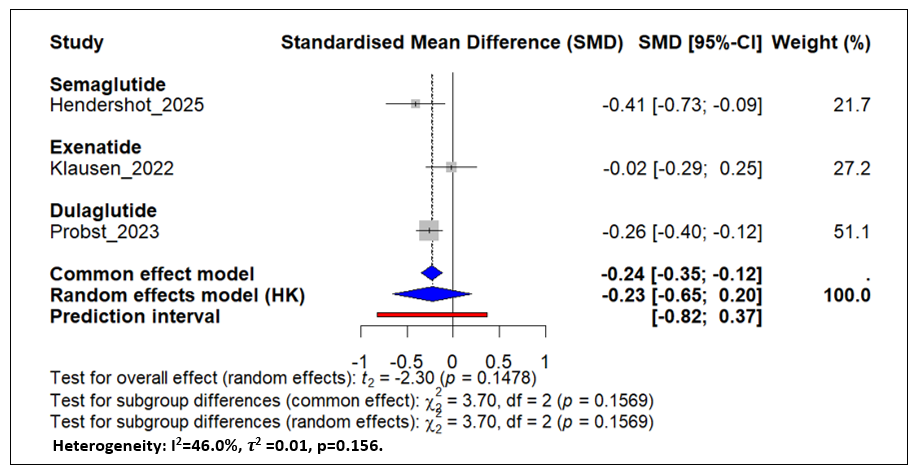


**Supplementary Figure S5:** Forest plot of the **subgroup analysis by type of glucagon-like peptide-1 receptor agonist (GLP-1RA) used** (semaglutide, exenatide) for the effect of GLP-1RAs on alcohol craving (measured via the **Penn Alcohol Craving Scale [PACS]) in randomised controlled trials (RCTs)**. The analysis includes two studies: Hendershot et al. (2025; semaglutide, SMD: -0.39, 95% CI: -0.73 to -0.05) and Klausen et al. (2022; exenatide, SMD: 0.01, 95% CI: -0.08 to 0.10). The diamond represents the pooled standardised mean difference (SMD) for each subgroup, with an overall pooled SMD of -0.16 (95% CI: -2.66 to 2.35, p = 0.024) using a random-effects model with the Hartung-Knapp (HK) adjustment. Negative SMD values favour GLP-1RAs over control. Heterogeneity is reported as I² = 80.3%, τ² = 0.064, p = 0.024, with a prediction interval (PI) of -4.24 to 3.92. The test for subgroup differences was significant (p = 0.024).


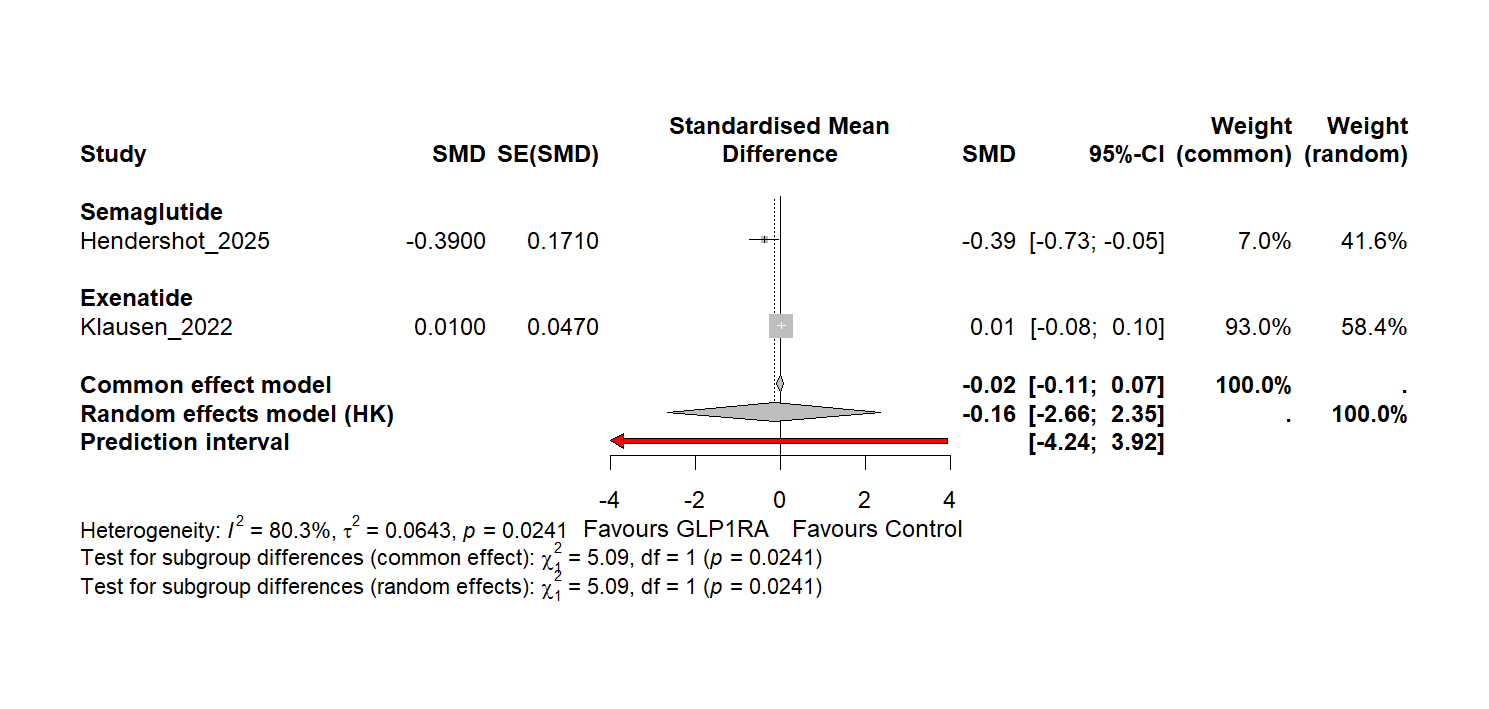


**Supplementary Figure S6**: Forest plot of the **sensitivity analysis for the effect of glucagon-like peptide-1 receptor agonists (GLP-1RAs) on total alcohol consumption in randomised controlled trials (RCTs),** excluding Probst et al. (2023) due to self-reported outcome bias. The analysis includes two studies: Hendershot et al. (2025) and Klausen et al. (2022). The diamond represents the pooled standardised mean difference (SMD) using a random-effects model with Hartung-Knapp (HK) adjustment, with a 95% confidence interval (CI) (SMD: -0.23, 95% CI: -3.15, 2.68, p = 0.496). Negative SMD values favour GLP-1RAs over control. Heterogeneity is reported as I² = 74.2%, τ² = 0.078, p = 0.049, with a prediction interval (PI) of -4.83 to 4.37.
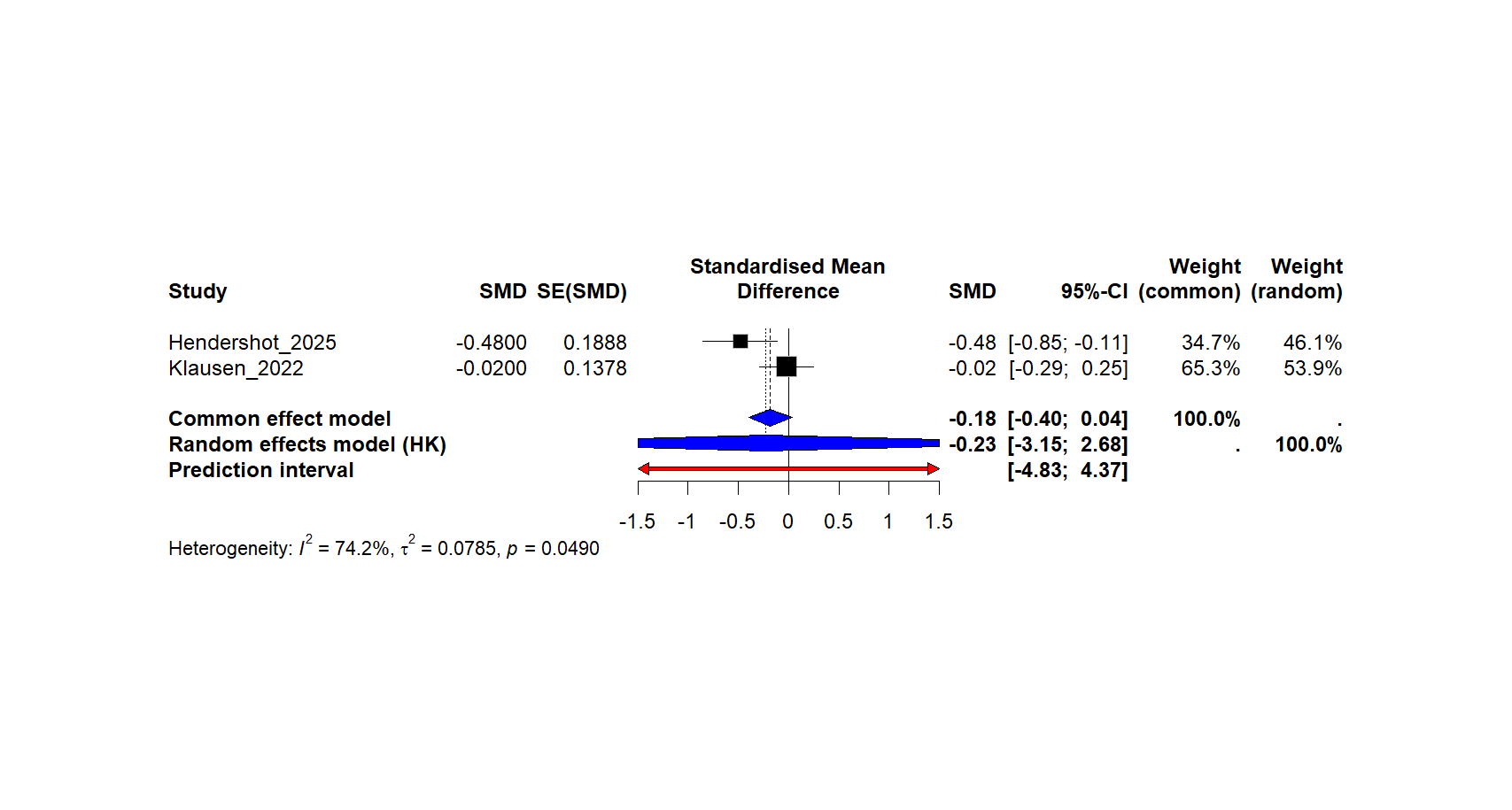


**Supplementary Figure S7:** **Funnel plot** assessing publication bias in the meta-analysis of **randomised controlled trials** (RCTs) evaluating the effect of glucagon-like peptide-1 receptor agonists (GLP-1RAs) on total alcohol consumption. The plot includes three studies: Hendershot et al. (2025), Klausen et al. (2022), and Probst et al. (2023). The x-axis represents the standardised mean difference (SMD), and the y-axis represents the standard error. The dashed lines indicate the expected distribution of studies in the absence of publication bias. The trim-and-fill method was not applied, and Egger’s test was not conducted due to the small number of studies (k < 10).
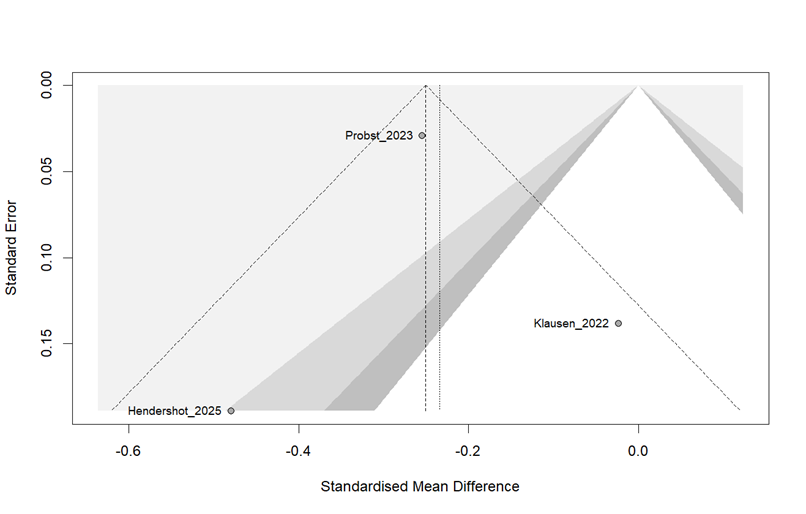


**Supplementary Figure S8:** Forest plot of the **subgroup analysis by outcome type (Alcohol Use Disorder [AUD] vs. Substance Use Disorder [SUD]) for the effect of glucagon-like peptide-1 receptor agonists (GLP-1RAs) on alcohol-related events**, measured as hazard ratios (HR) or adjusted HR (aHR). The analysis includes 11 estimates from six observational studies: Lähteenvuo et al. (2024; 4 estimates), Wium-Andersen et al. (2022; 2 estimates), Wang et al. (2024; 2 estimates), Qeadan et al. (2025; 1 estimate), Farokhnia et al. (2025; 1 estimate), and Xie et al. (2025; 1 estimate). The AUD subgroup (9 estimates, N = 1,447,840) yielded a pooled HR of 0.67 (95% CI: 0.62, 0.72, I² = 28.6%, τ² = 0.005, p = 0.190), and the SUD subgroup (2 estimates from Lähteenvuo et al., 2024; N = 227,868) yielded a pooled HR of 0.65 (95% CI: 0.48, 0.88, I² = 22.9%, τ² = 0.005, p = 0.254). The diamond represents the pooled HR for each subgroup, calculated using a random-effects model with the Hartung-Knapp (HK) adjustment. HR values below 1 favour GLP-1RAs over control. The test for subgroup differences was not significant (p = 0.793). Qeadan et al.’s intoxication outcome (HR = 0.50) is excluded from these panels but included in the overall pooled analysis (Figure 3) and reported narratively in Section 3.3.6.


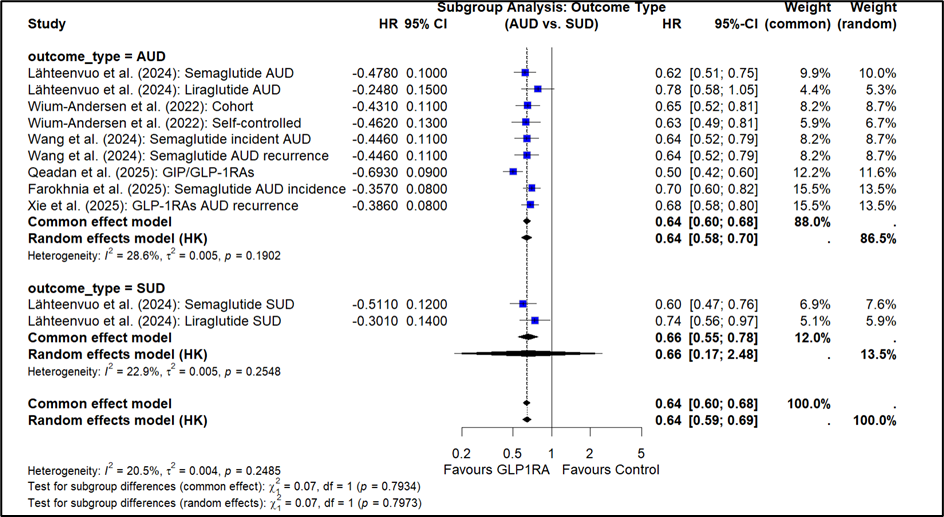


**Supplementary Figure S9:** Forest plot of the **subgroup analysis by type of glucagon-like peptide-1 receptor agonist (GLP-1RA) used (Semaglutide, Liraglutide, GIP/GLP-1RAs, Mixed) for the effect of GLP-1RAs on alcohol-related events**, measured as hazard ratios (HR) or adjusted HR (aHR), in observational studies. The analysis includes 11 estimates from six studies: Lähteenvuo et al. (2024; 4 estimates: Semaglutide AUD, Liraglutide AUD, Semaglutide SUD, Liraglutide SUD), Wium-Andersen et al. (2022; 2 estimates: Mixed), Wang et al. (2024; 2 estimates: Semaglutide), Qeadan et al. (2025; 1 estimate: GIP/GLP-1RAs), Farokhnia et al. (2025; 1 estimate: Semaglutide), and Xie et al. (2025; 1 estimate: Mixed). Subgroup results are: Semaglutide (5 estimates, HR: 0.65, 95% CI: 0.60, 0.70, I² = 0%), Liraglutide (2 estimates, HR: 0.76, 95% CI: 0.54, 1.06, I² = 0%), GIP/GLP-1RAs (1 estimate, HR: 0.50, 95% CI: 0.42, 0.60), and Mixed GLP-1RAs (3 estimates, HR: 0.66, 95% CI: 0.60, 0.73, I² = 0%). The diamond represents the pooled HR for each subgroup, calculated using a random-effects model with the Hartung-Knapp (HK) adjustment. HR values below one favour GLP-1RAs over control. The test for subgroup differences was significant (p < 0.001).


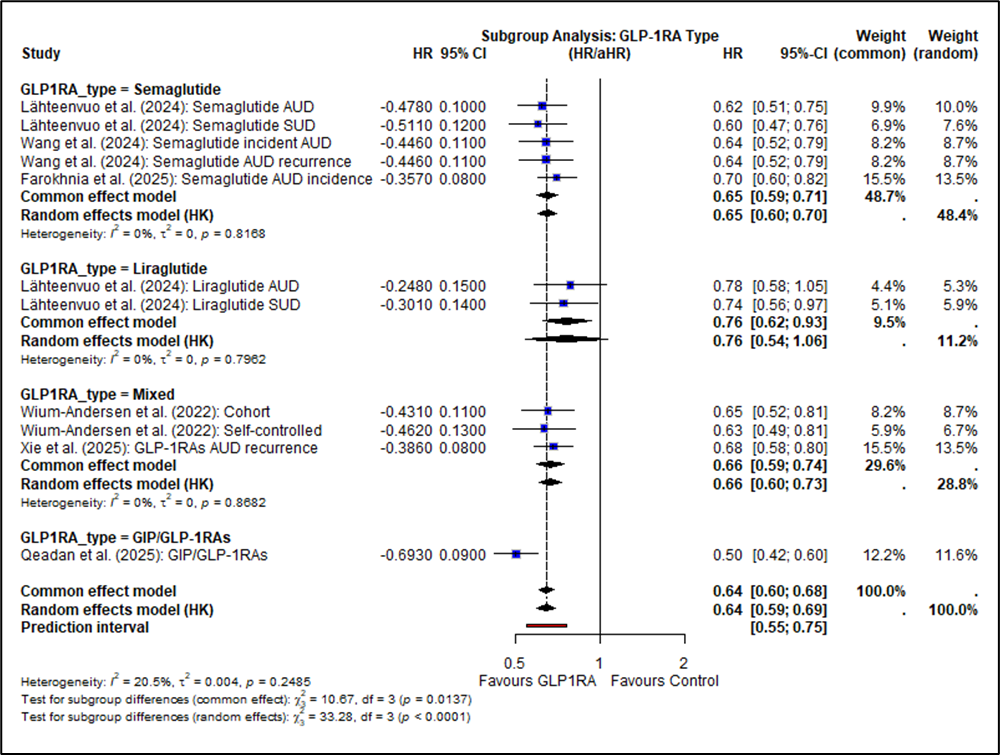


**Supplementary Figure S10:** Forest plot of the **sensitivity analysis for the effect of glucagon-like peptide-1 receptor agonists (GLP-1RAs) on alcohol-related events in observational studies.** The analysis includes 11 estimates from six studies: Lähteenvuo et al. (2024; 4 estimates), Wium-Andersen et al. (2022; 2 estimates), Wang et al. (2024; 2 estimates), Qeadan et al. (2025; 1 estimate), Farokhnia et al. (2025; 1 estimate), and Xie et al. (2025; 1 estimate). No studies were excluded as all had Newcastle-Ottawa Scale scores ≥7. The diamond represents the pooled hazard ratio (HR) obtained using a random-effects model with the Hartung-Knapp (HK) adjustment, along with a 95% confidence interval (CI) (HR: 0.64, 95% CI: 0.59-0.69, p < 0.001). HR values below 1 favour GLP-1RAs over control. Heterogeneity is reported as I² = 20.5%, τ² = 0.004, p = 0.248.


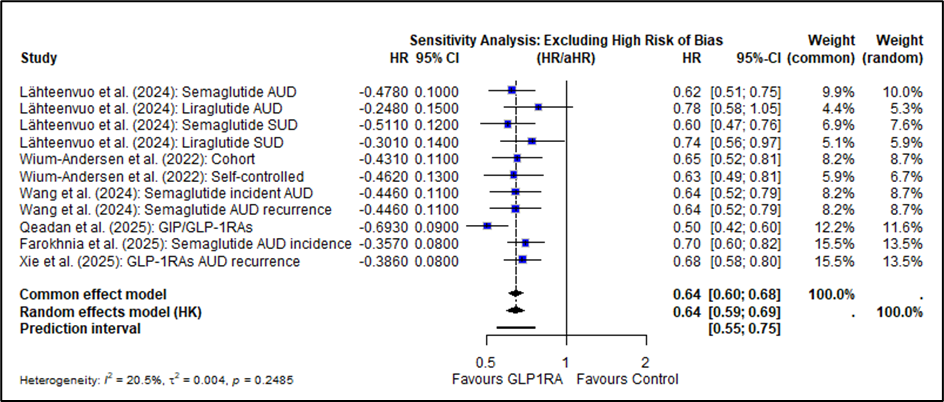


**Supplementary Figure S11:** **Funnel plot** assessing publication bias in the meta-analysis of **observational studies** evaluating the effect of glucagon-like peptide-1 receptor agonists (GLP-1RAs) on alcohol-related events. The plot includes 11 estimates from six studies: Lähteenvuo et al. (2024; 4 estimates), Wium-Andersen et al. (2022; 2 estimates), Wang et al. (2024; 2 estimates), Qeadan et al. (2025; 1 estimate), Farokhnia et al. (2025; 1 estimate), and Xie et al. (2025; 1 estimate). The x-axis represents the log hazard ratio (log HR), and the y-axis represents the standard error. The dashed lines indicate the expected distribution of studies in the absence of publication bias. Egger’s test showed no significant asymmetry (p = 0.555).

1: Lähteenvuo et al. (2024): Semaglutide AUD

2: Lähteenvuo et al. (2024): Liraglutide AUD

3: Lähteenvuo et al. (2024): Semaglutide SUD

4: Lähteenvuo et al. (2024): Liraglutide SUD

5: Wium-Andersen et al. (2022): Cohort

6: Wium-Andersen et al. (2022): Self-controlled

7: Wang et al. (2024): Semaglutide incident AUD

8: Wang et al. (2024): Semaglutide AUD recurrence

9: Qeadan et al. (2025): GIP/GLP-1RAs

10: Farokhnia et al. (2025): Semaglutide AUD incidence

11: Xie et al. (2025)


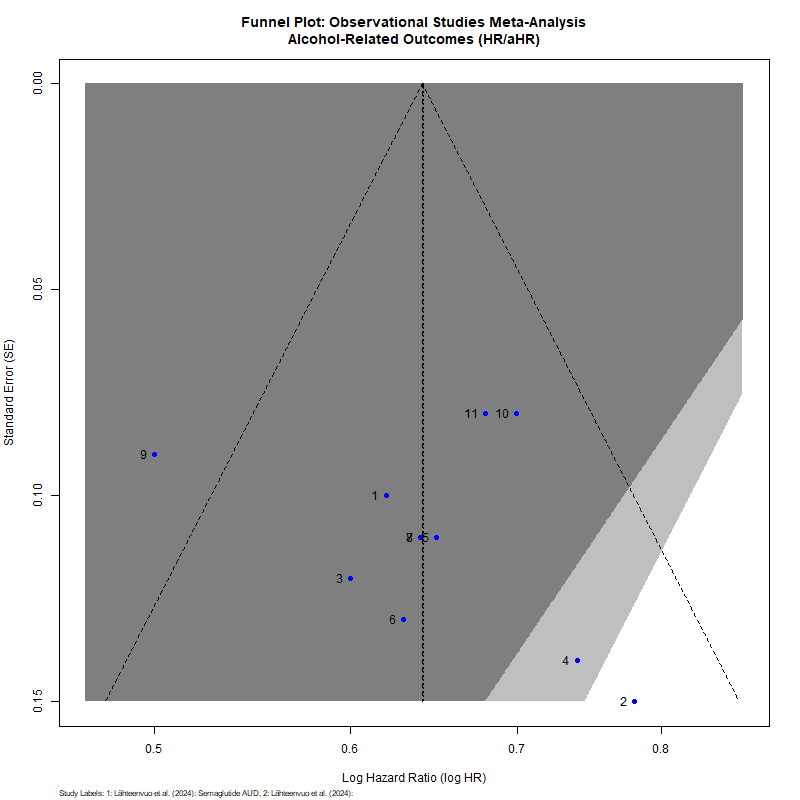


**Supplementary Figure S12:** Subgroup Analysis of AUD Outcomes by GLP-1RA Type (Excluding Qeadan et al.). Forest plot showing the effect of glucagon-like peptide-1 receptor agonists (GLP-1RAs) on alcohol use disorder (AUD) outcomes, stratified by GLP-1RA type (Semaglutide: k = 4, HR = 0.66, 95% CI: 0.60–0.72, I² = 0.0%; Liraglutide: k = 1, HR = 0.78, 95% CI: 0.58–1.05; Mixed GLP-1RAs: k = 3, HR = 0.66, 95% CI: 0.60–0.73, I² = 0.0%). The random-effects model with Restricted Maximum Likelihood (REML) estimation and Hartung-Knapp (HK) adjustment was used. No significant subgroup differences were found (p = 0.532). HR values below 1 favour GLP-1RAs over the control.

**
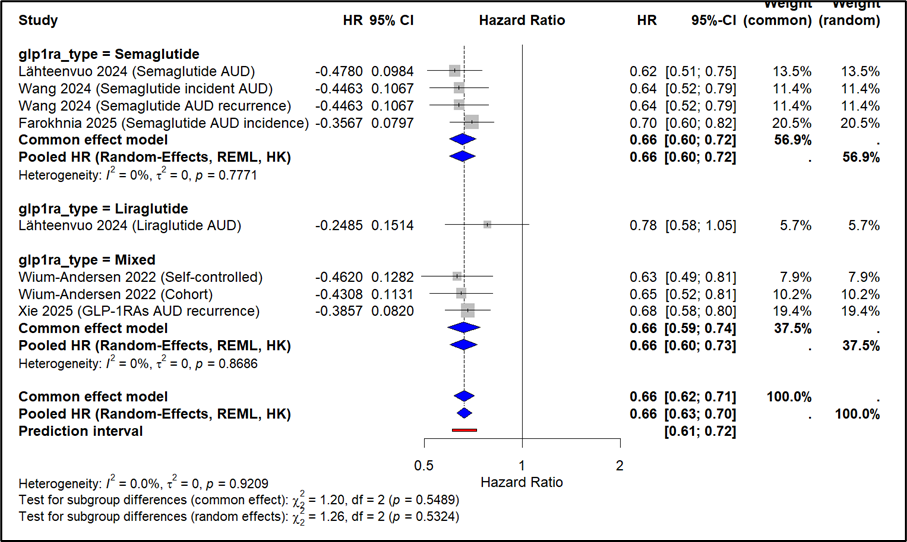
**

**Supplementary Figure S13:** Subgroup Analysis of SUD Outcomes by GLP-1RA Type. Forest plot showing the effect of glucagon-like peptide-1 receptor agonists (GLP-1RAs) on substance use disorder (SUD) outcomes, stratified by GLP-1RA type (Semaglutide: k = 1, HR = 0.60, 95% CI: 0.47–0.76; Liraglutide: k = 1, HR = 0.74, 95% CI: 0.56–0.97). The random-effects model with REML estimation and HK adjustment was used. Statistical comparison of subgroups was not feasible due to the single-study nature of the subgroups. HR values below 1 favour GLP-1RAs over the control.

**
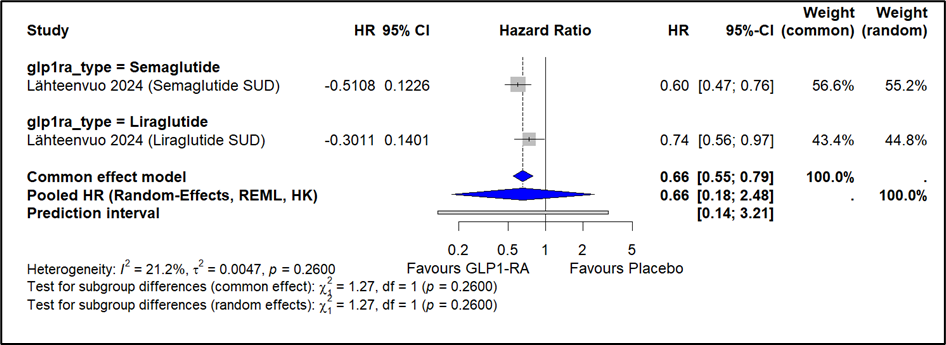
**

**Supplementary Figure S14**: Funnel Plot for AUD Outcomes (Excluding Qeadan et al.). Contour-enhanced funnel plot for the meta-analysis of alcohol use disorder (AUD) outcomes (k = 8 estimates), plotting log hazard ratios against standard errors. Contours represent significance levels (p > 0.10, p < 0.05, p < 0.01). Visual inspection shows no clear asymmetry, suggesting no evidence of publication bias. Egger’s test was not conducted due to k < 10.

**
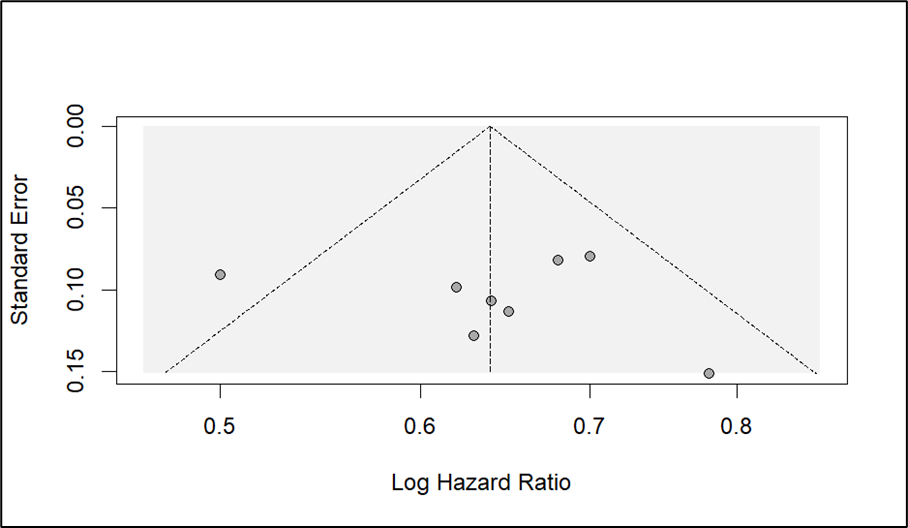
**

**Supplementary Figure S15:** Funnel Plot for SUD Outcomes. Contour-enhanced funnel plot for the meta-analysis of substance use disorder (SUD) outcomes (k = 2 estimates), plotting log hazard ratios against standard errors. Contours represent significance levels (p > 0.10, p < 0.05, p < 0.01). Visual inspection is limited by k = 2, and Egger’s test was not conducted due to insufficient studies.

**
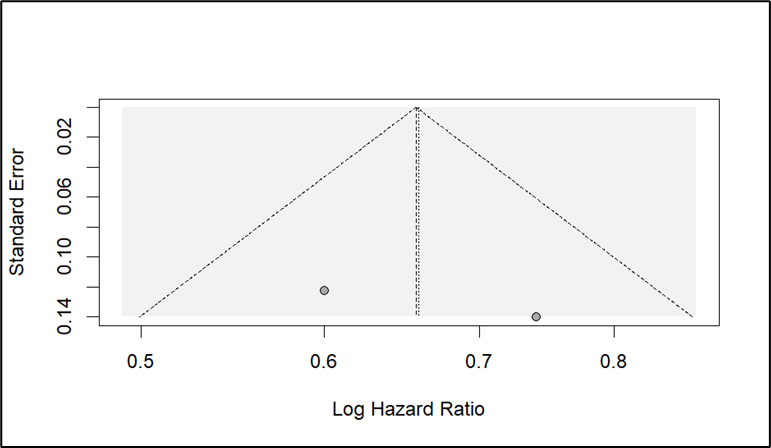
**
